# Cross center single-cell RNA sequencing study of the immune microenvironment in rapid progressing multiple myeloma

**DOI:** 10.1038/s41525-022-00340-x

**Published:** 2023-01-26

**Authors:** William Pilcher, Beena E. Thomas, Swati S. Bhasin, Reyka G. Jayasinghe, Lijun Yao, Edgar Gonzalez-Kozlova, Surendra Dasari, Seunghee Kim-Schulze, Adeeb Rahman, Jonathan Patton, Mark Fiala, Giulia Cheloni, Taxiarchis Kourelis, Madhav V. Dhodapkar, Ravi Vij, Shaadi Mehr, Mark Hamilton, Hearn Jay Cho, Daniel Auclair, David E. Avigan, Shaji K. Kumar, Sacha Gnjatic, Li Ding, Manoj Bhasin

**Affiliations:** 1 Aflac Cancer and Blood Disorders Center, Atlanta, GA USA; 2 Coulter Department of Biomedical Engineering, Emory University, Atlanta, GA USA; 3 Department of Pediatrics, Emory School of Medicine, Atlanta, GA USA; 4 Department of Medicine, Washington University School of Medicine, Saint Louis, MO USA; 5 Human Immune Monitoring Center, Icahn School of Medicine at Mt. Sinai, New York, NY USA; 6 Department of Genetics and Genomic Sciences, Icahn School of Medicine at Mount Sinai, New York, NY USA; 7 Division of Biomedical Statistics & Informatics, Department of Quantitative Health Sciences, Mayo Clinic, Rochester, MN USA; 8 Department of Oncological Sciences, Icahn School of Medicine at Mount Sinai, New York, NY USA; 9 Beth Israel Deaconess Medical Center, Harvard Medical School, Boston, MA USA; 10 Mayo Clinic Rochester, Division of Hematology, Rochester, MN USA; 11 Department of Hematology/Medical Oncology Emory University School of Medicine, Atlanta, GA USA; 12 Winship Cancer Institute, Emory University, Atlanta, GA USA; 13 Washington University School of Medicine, St Louis, MO USA; 14 Multiple Myeloma Research Foundation (MMRF), Norwalk, CT USA; 15 Department of Biomedical Informatics, Emory School of Medicine, Atlanta, GA USA

**Keywords:** Prognostic markers, Myeloma

## Abstract

Despite advancements in understanding the pathophysiology of Multiple Myeloma (MM), the cause of rapid progressing disease in a subset of patients is still unclear. MM’s progression is facilitated by complex interactions with the surrounding bone marrow (BM) cells, forming a microenvironment that supports tumor growth and drug resistance. Understanding the immune microenvironment is key to identifying factors that promote rapid progression of MM. To accomplish this, we performed a multi-center single-cell RNA sequencing (scRNA-seq) study on 102,207 cells from 48 CD138^-^ BM samples collected at the time of disease diagnosis from 18 patients with either rapid progressing (progression-free survival (PFS) < 18 months) or non-progressing (PFS > 4 years) disease. Comparative analysis of data from three centers demonstrated similar transcriptome profiles and cell type distributions, indicating subtle technical variation in scRNA-seq, opening avenues for an expanded multicenter trial. Rapid progressors depicted significantly higher enrichment of *GZMK*^*+*^ and *TIGIT*^+^ exhausted CD8^+^ T-cells (*P* = 0.022) along with decreased expression of cytolytic markers (*PRF1, GZMB, GNLY*). We also observed a significantly higher enrichment of M2 tolerogenic macrophages in rapid progressors and activation of pro-proliferative signaling pathways, such as BAFF, CCL, and IL16. On the other hand, non-progressive patients depicted higher enrichment for immature B Cells (i.e., Pre/Pro B cells), with elevated expression for markers of B cell development (*IGLL1*, *SOX4*, *DNTT*). This multi-center study identifies the enrichment of various pro-tumorigenic cell populations and pathways in those with rapid progressing disease and further validates the robustness of scRNA-seq data generated at different study centers.

## Introduction

Multiple myeloma (MM) is one of the most frequent hematological cancers, representing 10% of hematological malignancies in the US, and 1.7% of malignancies overall^1^. Though significant advances have been made in MM therapeutics, such as the development of immunomodulatory drugs like lenalidomide and proteasome inhibitors (bortezomib), MM remains incurable. The prognosis following diagnosis remains poor, with a median survival of four to five years after treatment^2^. The bone marrow (BM) microenvironment is altered by the myeloma cells to support proliferation, drug resistance, and immune evasion^3–5^. MM patients commonly relapse following initial treatment, after which the prognosis notably worsens, and patients eventually succumb to their disease^6^.

Given the significance of the BM microenvironment (BME) in the progression of MM, the understanding of what factors comprise anti- or pro-tumor roles in the BME is critical for developing the next generation of MM therapies. Genomics studies have shown that complex interactions among genetic variants, gene fusions, and translocations together with epigenetics, translate into heterogeneous outcomes and kinetics of disease progression^7–9^. The accumulating evidence shows that the composition and expression profiles of immune and stromal cells in BME are significantly associated with disease progression and therapeutic outcomes across solid and hematological cancers^2,6,10–14^. Immune cells of BME make up a crucial immunosuppressive pre-malignant niche with enrichment of regulatory T cells (Tregs), myeloid-derived suppressor cells (MDSCs), tumor-associated macrophages (TAMs), and scarcity of CD4^+^ or CD8^+^ T cells, and NK cells^2^.

Previous studies have identified disruptions that contribute to immune escape (angiogenesis, pro-proliferation, etc.) in the BME. Adhesion to BM stromal cells contributes to the release of angiogenic signals, such as VEGF, that promote tumor growth. Production of cytokines, such as IL-6, by the myeloma cells, have not only been associated with the enrichment of anti-apoptotic factors in myeloma cells but also impaired dendritic cell function and T-cell activation^13^. Alterations in the number of cells expressing a NK phenotype have been noted in the BM of MM patients^13^, along with impairment of these cells through the downregulation of activating receptors^15^. BM T-cells from MM patients with advanced disease show elevation of immune-inhibitory receptors and markers related to senescence, relative to healthy controls^16^. A recent single-cell RNA-sequencing (scRNA-seq) study has shown dysregulation of cytotoxic T cells and major histocompatibility complex class II expression in CD14+ monocytes over the course of progression from premalignant to symptomatic MM^11^. Collectively, all these disruptions in the BME form an environment that allows myeloma cells to thrive and aggressively proliferate.

In this study, we use scRNA-seq to compare the BME in BM aspirates taken at diagnosis from 18 MM patients classified as either ‘Rapid Progressors’ (RP; PFS < 18 months) or ‘Non-Progressors’ (NP; PFS > 4 years) as part of the MMRF CoMMpass study (Supplementary Table 1). We studied the overall changes in the immune cell composition within the BM of these patients, along with differentially expressed genes between the two groups for common cell types. In addition, technical biases in the scRNA-seq data were investigated by comparative analysis of scRNA-seq libraries prepared and sequenced from the same samples (aliquots) across three medical centers (Beth Israel Deaconess Medical Center, Boston (BIDMC), Washington University in St. Louis (WashU), and Mount Sinai School of Medicine, NYC (MSSM)). The technical evaluation of the single-cell assays depicted similar gene expression profiles of different cell types across three medical centers, demonstrating the feasibility of the multi-center trials. Comparative analysis of RP and NP showed significant enrichment of an exhausted T-cell population, associated with the expression of GZMK and decreased cytolytic markers (*PRF1, GZMB, GNLY*) in the rapid progressors. We also observed the enrichment of interferon- alpha and -gamma downstream targets in RP myeloid cells along with the enrichment of M2 tolerogenic macrophages. The cellular communication and interaction analysis depicted enriched communication of the BAFF and CCL signaling pathways in rapid progressors T and myeloid cells.

## Results

### Overview of the CD138^−^ cell fraction

ScRNA-seq data from the CD138^-^ cells fraction of 48 BM biopsies from 18 patients were combined into an integrated dataset of 102,207 single-cells generated at three different medical centers. Low-quality cells are filtered out by removing cells with <200 unique genes, <500 UMI reads, and >30% mitochondrial UMIs. The malignant plasma cells, identified based on marker genes expression (*MZB1*+*, JCHAIN*+*, SDC1*+) (Supplementary Fig. 1a, b), heterogeneity as well as CNV analysis, were filtered out. The filtering of low-quality and malignant plasma cells resulted in a final integrated dataset of 90,502 CD138^-^ single-cells.

**Fig. 1:**
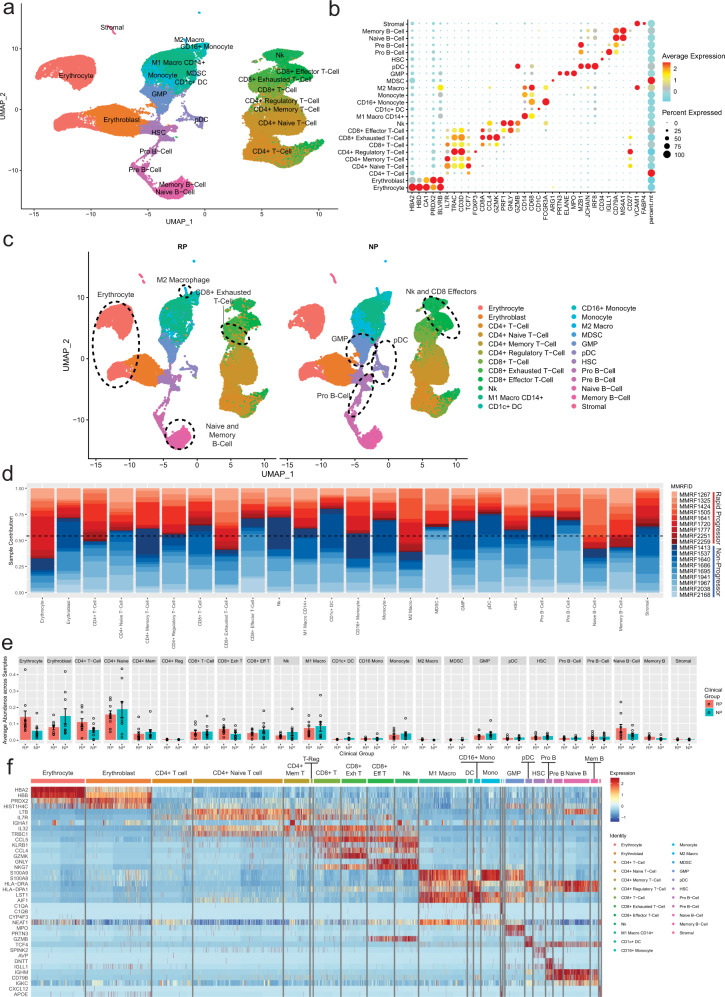
Single-cell profiling of bone marrow from MM patients with rapid (RP) and no progression (NP). Clinical samples with rapid and no progression were identified from the Multiple Myeloma Research Foundation (MMRF) CoMMpass study, a longitudinal genomic study of patients with newly diagnosed, active multiple myeloma (NCT01454297). In the study, 48 bone marrow aliquots from 18 patients diagnosed with Multiple Myeloma (MM) were processed for scRNA-seq at three medical centers, Beth Israel Deaconess Medical Center (BIDMC), Mount Sinai School of Medicine (MSSM), and Washington University (WashU). Patients are classified as either rapid progressors (RP) or non-progressors (NP) based on the rate of disease progression, <18 months or >4 years post-diagnosis, respectively. **a** Uniform manifold approximation and projection (UMAP) embedding of scRNA samples across all patients consisting of 90,502 high quality single-cells portioned into 24 cell types. Plasma cells were removed prior to embedding. These clusters are colored based on canonical cell types based on the expression of marker genes that include erythrocytes (*HBA*^*+*^*, HBD*^+^), erythroblasts (*BLVRB*^*+*^*, PRDX2*^+^), T-cells *(IL7R*^*+*^*, CD3D*^+^), CD8^+^ T-cells (*CD8A*^*+*^*, CCL4*^*+*^*, GZMK*^+^), CD8^+^ effector T-cells (*GNLY*^*+*^*, GZMB*^*+*^*, CD3D*^+^), NK Cells (*GNLY*^*+*^*, GZMB*^*+*^*, CD3D*^*-*^*)*, monocytes/macrophages *(CD14*+*, CD68*^*+*^*), CD1c*^*+*^ *DC (CD1c*^*+*^*)*, Granulocyte-Macrophage Progenitors-GMP (*ELANE*^*+*^*, MPO*^+^), plasmacytoid dendritic cells-pDC *(IRF8*^*+*^*, MZB1*^+^), HSC (*CD34*^*+*^), Pro B-Cells (*IGLL1*^*+*^), and B-cells (*MS4A1*^*+*^*, CD79A*^+^). **b** Dot Plot depicting expression profile of markers genes used for annotating different cell type clusters. The over and under expression of specific markers is shown by red and cyan colors, respectively. **c** Clinical phenotype based split UMAP showing the distribution of cell types in the RP and NP groups. There is slightly elevated NK, CD8^+^ effector, Pro B-Cell, GMP, and pDC counts in the NP group, while RP samples show elevation of exhausted T-cells, naive and memory B-cells, and erythrocytes. **d** A stacked bar plot showing the relative patient contribution to each individual cell type cluster. The patients from RP and NP groups are shown with shades of red and blue, respectively. Each cluster depicted the varying levels of contribution from multiple samples of NP and RP groups. **e** Comparative analysis of cell types enriched in the RP and NP groups. Each bar plot depicts the mean and ±standard error of the mean in NP and RP groups. Each dot represents an individual patient sample. **f** A heatmap displaying the top markers expressed by each cell type. Columns represent individual cells, grouped by cell type, while rows display individual genes. Horizontal colored bars above the heatmap indicate the cell type, with the legend on the right listing the cell type for each colored bar. Cell type labels are also displayed above their corresponding bar for all cell types except for the three smallest populations (M2 macrophages, MDSCs, and stromal cells). Relative gene expression is shown in pseudo color, where blue represents downregulation, and red represents upregulation. Top markers generally correlate with well-established canonical markers for each cell type.

Bone marrow cells were clustered based on gene expression profile (Fig. 1a) and annotated to cell types from various lineages using canonical marker genes: erythrocytes (*HBA*^*+*^*, HBD*^*+*^), erythroblasts (*BLVRB*^+^, *PRDX2*^+^), T-cells (*IL7R*^+^, *CD3D*^+^), CD8^+^ T-cells (*CD8A*^+^, *CCL4*^+^, *GZMK*^+^), CD8^+^ effector T-cells (*GNLY*^+^, *GZMB*^+^, *CD3D*^+^), NK Cells (*GNLY*^+^, *GZMB*^+^, *CD3D*^-^), monocytes/macrophages (*CD14*^+^, *CD68*^+^), CD1c^+^ dendritic cells (*CD1c*^+^), Granulocyte-Macrophage Progenitors-GMP (*ELANE*^+^, *MPO*^+^), plasmacytoid dendritic cells-pDC (*IRF8*^+^, *MZB1*^+^), hematopoietic stem cells-HSC (*CD34*^+^), Pro B-Cells (*IGLL1*^+^), and B-Cells (*MS4A1*^+^, *CD79A*^+^) (Fig. 1a, b). All the defined 24 cell types were detected in samples from both NP and RP groups (Fig. 1c). Most of the cell type clusters contained samples from multiple patients with none of the clusters being dominated exclusively by a single patient or sample (Fig. 1d). On average, CD4^+^ T-cells were the largest cluster among all patients, followed by erythrocytes, myeloid cells, CD8^+^ T-cells, and B-cells (Fig. 1e). Some cell types such as CD8^+^ exhausted T-cells and Memory B-cells are slightly elevated in RP, though currently these differences are not significant as a proportion of all cells. Further generation of unbiased cell type signature based on supervised analysis (*P* < 0.01, FC > 2) identified top markers that generally correspond with what is expected for each cell type (e.g., *GNLY* for CD8+ effector T-cells, *LTB* for CD4^+^ naive T-cells) (Fig. 1f). We observed overexpression of some immune markers such as *IGHA1* that correspond to samples from an individual patient and may represent contamination from apoptotic plasma cells. Additional more stringent mitochondrial cutoff (filtering out cells with >20% mitochondrial DNA content) analysis depicted similar clustering patterns across all cell types (Supplementary Fig. 2a) and within the T-cell, Myeloid, and B-cell compartments (Supplementary Figs. 3–5). We also observed a significantly higher ratio of exhausted T cells (*P* = 0.049) (Supplementary Fig. 3b) as well as a significant enrichment of M2 macrophages (*P* = 0.049) (Supplementary Fig. 4b) in the RP compared to the NP samples.

To assess the potential malignancy of our plasma cell population (Supplementary Fig. 1b), we performed copy number variation (CNV) analysis^17^ using either normal plasma cells from the Human Cell Atlas (HCA) Census of Immune Cells^18^ or the naive and memory B cell populations from the current study dataset as reference cells. The clustering and UMAP analysis depicted that plasma cells formed multiple patient clusters with no co-clustering with the mature B cell populations indicating heterogeneity and difference in transcriptomes profiles (Supplementary Fig. 6a). CNV analysis identified multiple amplifications and deletions in a patient-specific manner on several chromosomes as well as promiscuous deletions on chromosomes 1 and 6 in the plasma cells (Supplementary Fig. 6b). These deletions/amplifications in the plasma cells as compared to B cell populations likely correspond to the malignant phenotype of plasma cells. Further comparative analysis of plasma cells from this study with the normal plasma cells from HCA also depicted significant heterogeneity and CNVs pointing toward the malignant phenotype of plasma cells identified in this study (Supplementary Fig. 7).

### CD138^−^ microenvironment cells exhibited similar single-cell profiles for three different testing centers

To assess the center/sample processing technical variations in the single-cell profiles, samples were processed from the same patients at three different medical centers/universities (BIDMC, WashU, and MSSM). All the samples were processed with droplet-based single-cell barcoding techniques for scRNA-seq alone (WashU, MSSM) or CITE-Seq (BIDMC). Twenty samples from 18 patients were processed at BIDMC, seven samples from seven patients were processed at MSSM, and 21 samples from 17 patients were processed at WashU (Supplementary Table 1). Samples had similar age distributions between both RP and NP groups, however, RPs had higher risk assessments at diagnosis as defined by the IMWG risk class (Supplementary Table 1).

**Fig. 2:**
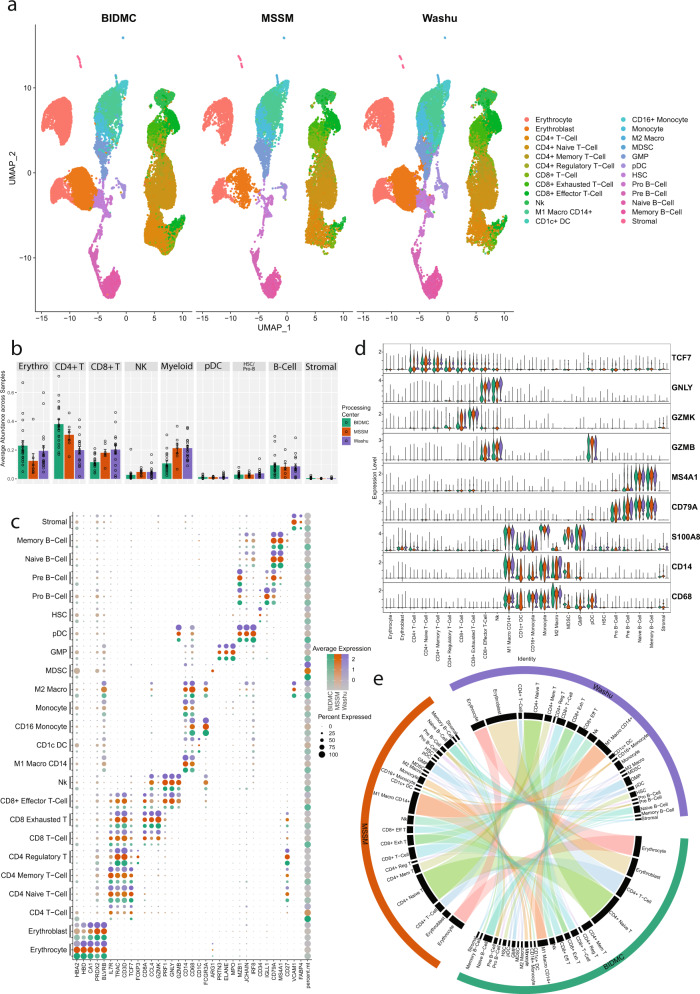
Comparison of scRNA profiles of samples processed at three different centers. Bone marrow aspirates from the same set of patients were processed at three different centers, BIDMC, MSSM, and WashU, and analyzed using a uniform bioinformatics workflow for comparative analysis. The comparative analysis was performed on 20 samples processed at BIDMC, 7 processed at MSSM, and 21 processed at WashU. **a** Split UMAP based on sample processing centers of scRNA samples. All major cell types are captured in the single-cell profile from each center. Clusters are colored based on cell types identified in Fig. 1a. **b** Comparative analysis of cell type proportion across centers. Each bar represents the mean ratio for a given cell type for all samples processed at a specific center. Error bars show the standard error of the mean. Individual dots represent individual patient cell type ratios. Samples from MSSM and WashU had similar ratios across the cell types. BIDMC shows a higher proportion of CD4^+^ T-cells and a lower ratio of Myeloid cells. **c** Comparative analysis of canonical cell type-specific markers across three centers. Most of the cell type defining markers are concordantly expressed across cell types indicating strong similarity in the single-cell profiles generated across centers. BIDMC, which performed CITE-Seq, tends to have higher percent.mt relative to other centers this might be due to longer processing time for CITE-Seq due to antibody labeling. **d** Violin Plots comparing the expression of various cell markers among different centers. Overall, the level of expression of these markers are consistent among centers indicating no batch effect or center-based expression artifact. **e** A Circos plot showing the correlation between expression profiles of cell types profiled at different centers. The individual cell types across centers depict significant similarity in the expression profiles. Some cell types with lower correlations include CD4^+^ memory T-cells from BIDMC, and monocytes and CD1c^+^ DCs from WashU.

Prior to the batch correction, most of the cell types from the three centers depicted similar clustering patterns with subtle variations among processing centers (Supplementary Fig. 8a). Shannon’s entropy was computed per cell to assess the degree of mixing of samples from centers, and low entropy values, indicating poor mixing, can be observed in MSSM samples, and in the ‘CD4^+^ T-cell’ class from BIDMC (Supplementary Fig. 9b, d). The batch effect correction improved the entropy values indicating better mixing of cells from different centers (Supplementary Fig. 9c, e). The evaluation of batch effect and corrections in the other cellular compartments (monocytes/macrophages, B cells) also depicted that batch effect correction improved the mixing of cell types/subtypes from different processing centers effectively alleviating site-dependent batch effect (Supplementary Figs. 10–11). All major cell types (Fig. 1a) are uniformly detected in samples processed at each center as well as co-embed in the clustering indicating similarity in transcriptome profiles (Fig. 2a). This includes even rare cell populations such as the stromal and CD4^+^ regulatory T-cells, which represent <1% of the total cells profiled. Samples from MSSM and WashU had a similar ratio for all cell types. The comparative analysis of cellular proportions among the centers depicted subtle variation, which might be due to differences in the number of cells captured for single-cell assay, sequencing depth, and assays performed. All three centers captured striking similar proportions of pDCs, HSCs, B cells, and stromal cells (Fig. 2b). The samples processed at BIDMC depicted variation in the proportion of T/NK and myeloid cells compared to MSSM and WashU.

Samples processed at BIDMC showed a higher proportion of CD4^+^ T-cells and a lower proportion of myeloid cells relative to samples processed at other centers (Fig. 2b). Some of these differences may be explained by the additional processing time required for BIDMC samples (simultaneous measurement of surface marker expression along with gene expression via the CITE-seq approach). A high proportion of the CD4^+^ T-cells in the BIDMC group belong to the general “CD4^+^ T-cell” clusters (Supplementary Fig. 12a, b), which tend to consist of lower viability samples with higher mitochondrial reads compared to other clusters. Furthermore, the comparative analysis of cellular subtypes for myeloid and B cells depicted striking similarities among the three centers (Supplementary Fig. 12c, d).

Further correlation of cell type proportions of individual samples processed at different centers depicted no specific pattern (Supplementary Fig. 13). To further explore the impact of sample processing from different centers on gene expression, we performed a comparative gene expression profile analysis of cell types and subtypes obtained from the three centers (Supplementary Fig. 14). Without batch correction, correlation is primarily driven by the cell compartment over the processing center, with T-cells, myeloid cells, B cells, and Erythroid cells clustering strongly regardless of the processing center. Within these compartments, there is a similar pattern as observed in the uncorrected embeddings, that is that WashU and BIDMC samples tend to have higher correlations relative to MSSM samples. MDSCs, a very small myeloid population, show the largest differences between centers. This appears to be driven by the lower viability of this cluster based on mitochondrial expression, along with a low cell count.

Next, we assessed the similarity in canonical markers expression for various centers. The canonical markers for each cell type depicted similar expressions for most cell types (Fig. 2c), with the primary exception being the previously noted MDSC cluster. The consistency of key marker expression ensures that cell types can be identified reliably across all three centers, and comparative analysis can be performed among the samples generated at different centers. This is further evident in violin plots of select markers illustrating the consistency in both the average expression and the distribution of gene expression within a cell population (Fig. 2d). The primary difference in gene expression between the centers is higher percentages of mitochondria-associated transcripts from samples processed at BIDMC (Supplementary Fig. 15). This may be attributed to a decrease in cell viability associated with the longer processing times for cell surface protein labeling before proceeding with the single-cell gene expression library preparation protocols at BIDMC.

To further validate the consistency of cell type labeling across centers, we assessed the similarity of the differentially expressed markers for each of the 24 cell types across each center. To achieve this, cells from each center were subset, and the top differentially expressed markers for each cell type with respect to all other cells from the same center were identified. Cell types from each center were clustered hierarchically based on the binary distance between these differentially expressed markers (Supplementary Fig. 16). We observed that overall, the differentially expressed cell type markers from all three centers strongly correlated with the matching cell type from other centers. This includes closely related cell types such as CD8^+^ T-cells and CD8^+^ exhausted T-cells (Fig. 2e). A few cell types, e.g., CD4^+^ Memory T-cells from BIDMC, HSCs, and Erythroblasts from MSSM, and certain myeloid cell types, such as CD1C^+^ DC, monocytes, and MDSCs, showed weaker correlation between centers. Some of these weaker correlations may be driven by large contributions from individual patient samples processed at certain centers. For example, a relatively large proportion of CD4^+^ Memory T-cells from BIDMC are from MMRF1413, which has a higher degree of plasma cell contamination, notably IGHA1, relative to other samples (Supplementary Fig. 17). In summary, the single-cell profiles generated at three different centers with different approaches have considerable similarities in their overall gene expression profiles. These results demonstrate that center-independent analysis can be implemented for a large cohort of samples processed at different centers after implementing appropriate batch correction approach.

### Rapid progressors depicted significant enrichment of T cell exhaustion and attenuation of CD8^+^ effector T-cells

**Fig. 3:**
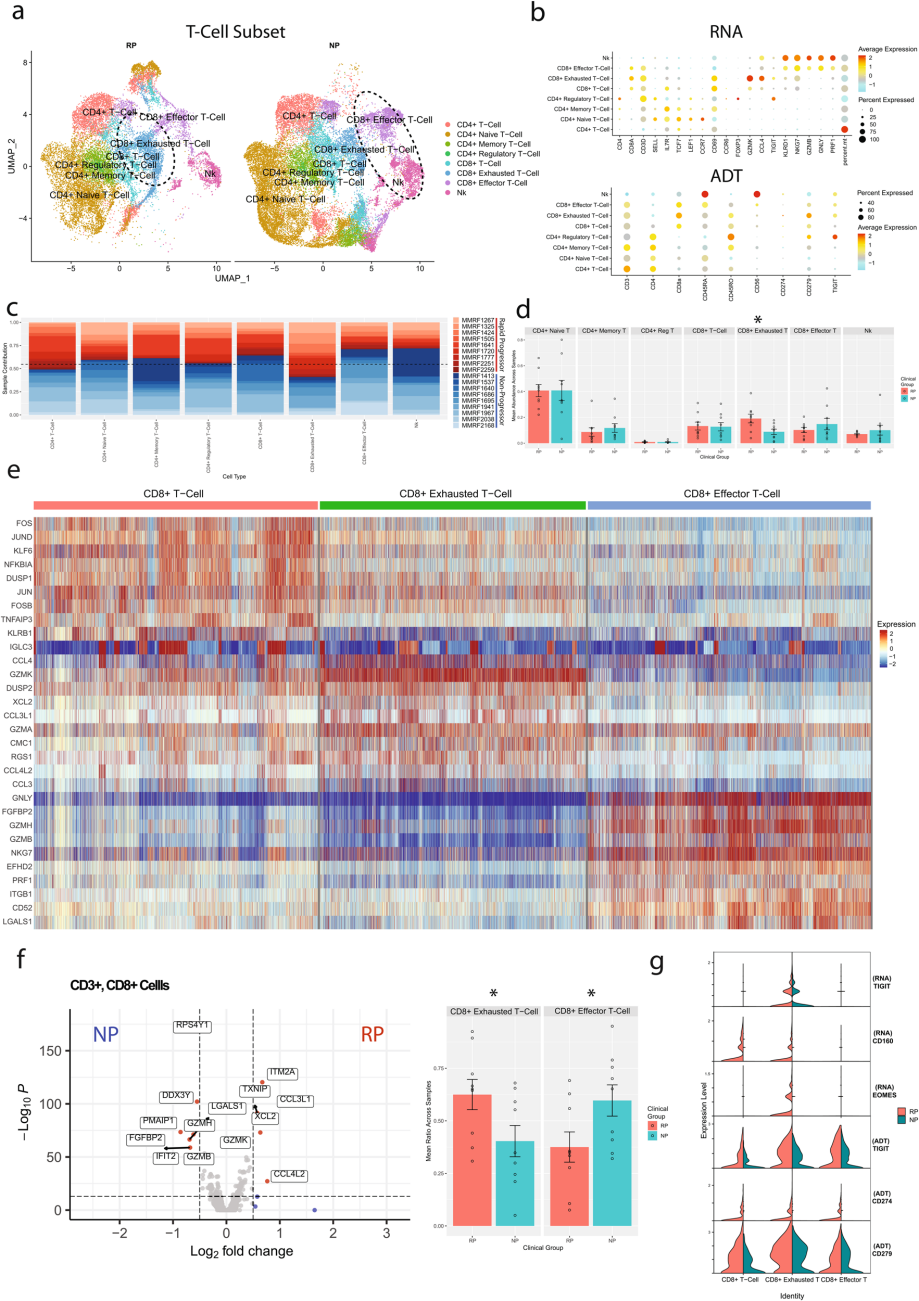
Comparative analysis of T and NK cell subpopulations in multiple myeloma patients with rapid- and no- progression of the disease. **a** A UMAP displaying the T-cell subclusters split based on clinical groups (i.e., NP, RP). Subclusters were manually labeled as CD4^+^ T-cells (naive, memory, regulatory), CD8^+^ T-cells (memory, exhausted, effector), or NK cells based on the expression of specific markers. Limited CITE-Seq data from BIDMC was used to confirm some cellular annotations. NK and CD8^+^ effector T-cells show elevated counts from NP samples, while RP samples contain higher counts of CD8^+^ exhausted T-cells. **b** Dot plot demonstrating the expression profile of key markers for each T-cell subtype from both the scRNA-seq and CITE-Seq (ADT) assays. Markers for cell types used were CD4^+^ T-cells (CD4^+^), CD4^+^ naive T-cells (*CD4*^+^, *TCF7*^+^, *CCR7*^+^, CD45RO^+^), CD4^+^ Memory T-cells (CD4^+^,*IL7R*^+^, CD45RA^+^), CD4^+^ regulatory T-cells (CD4^+^, *FOXP3*^+^), CD8^+^ T-cell (CD8A^+^, *KLRB1*^+^, *IL7R*^+^, *GATA3*^+^, *GZMK* low), CD8^+^ exhausted T-cells (*CD8A*^+^, *GZMK*^+^, *TIGIT*^+^), CD8^+^ effector T-cells (*CD3D*^+^, *GNLY*^+^, *GZMB*^+^), and NK cells (*CD3D*^-^, *GNLY*^+^, *GZMB*^+^). **c** The patient contribution to each cell type cluster indicates that most of the clusters consist of cells from multiple patients. The patients from RP and NP groups are shown with shades of red and blue respectively. **d** Comparative analysis of the T-cell types enriched in the RP and NP T-cell subsets. Each bar plot depicts mean and standard error of mean. Significant enrichment (*P* = 0.022) of the CD8^+^ exhausted T-cells was observed in the RP population. **e** Differential expression analysis of the three CD8^+^ T-cell subtypes (CD8^+^ memory T-cells, CD8^+^ exhausted T-cells, CD8^+^ effector T-cells). Columns represent individual cells, grouped by cell type, while rows display individual genes. CD8^+^ T-cells depicted upregulation of markers related to T-cell memory, such as IL7R, but has under-expression of cytotoxic markers such as *GZMK*, *GZMB*, or *NKG7* as compared to other CD8 T-cell subtypes. CD8^+^ exhausted T-cells depicted upregulation of *GZMK* and multiple genes related to chemokine signaling, such as *CCL3* and *XCL2*. CD8^+^ effector T-cells showed downregulation of *GZMK* and upregulation of *GZMB* and *GZMH*, along with cytotoxic markers such as *PRF1* and *GNLY*. **f** Comparative analysis of the CD8^+^ subset between NP and RP groups. Differential expression analysis was performed based on the Wilcoxon rank sum test of NP and RP CD8^+^ T-cells. NP CD8^+^ T-cells showed upregulation of markers related to NK cells, such as *GZMB* and *GZMH*. RP CD8^+^ T-cells instead show upregulation of the CD8^+^ exhausted T-cell markers, specifically chemokines like *CCL3L1* and *XCL2*. These differences are reflected in the average cell type ratios in the CD8+ subset in NP and RP samples. The ratio of these CD8^+^ exhausted T-cells to the GZMB^+^ effector cells is significantly higher in RP samples (*P* = 0.048). **g** Expression profile of markers of exhaustion in the CD8^+^ and NK subsets. CD8^+^ exhausted T-cells, predominantly found in the RP group, show the highest expression of *TIGIT* and *EOMES* in the RNA assay relative to other CD8^+^ T-cells. *CD160* is detectable in CD8^+^ T-cells and CD8^+^ exhausted T-cells, though only in samples from the RP population. CITE-Seq confirms elevated expression of TIGIT and PD-1 (CD279) in the CD8+ exhausted T-cell cluster, and general enrichment of exhaustion markers in the RP group over the NP group across multiple CD8+ cell types.

Focused analysis was carried out on T-lymphocyte and NK cells to understand their role in the progression of MM (rapid or non-progression). The analysis included 43,039 cells; 25,381 cells from NP samples and 17,658 cells from RP samples, which were identified as eight different subtypes of T and NK cells following further clustering (Fig. 3a). These cells were classified based on RNA and protein/ADT data: CD4^+^ T-cells (*CD4*^*+*^), CD4^+^ naive T-cells (*CD4*^*+*^*, TCF7*^*+*^*, CCR7*^*+*^*, CD45RO*^*+*^), CD4^+^ memory T-cells (*CD4*^*+*^*,IL7R*^*+*^*, CD45RA*^*+*^), CD4^+^ regulatory T-cells (*CD4*^*+*^*, FOXP3*^*+*^), CD8^+^ T-cell (*CD8A*^*+*^*, KLRB1*^*+*^*, IL7R*^*+*^*, GATA3*^*+*^*, GZMK* low), CD8^+^ exhausted T-cells (*CD8A*^*+*^*, GZMK*^*+*^*, TIGIT*^*+*^), CD8^+^ effector T-cells (*CD3D*^*+*^*, GNLY*^*+*^*, GZMB*^*+*^), and NK cells (*CD3D*^-^, *GNLY*^*+*^, *GZMB*^*+*^) (Fig. 3b).

In both NP and RP groups, various subtypes of CD4^+^ T-cells were the dominant T-cells (~60%), with the remaining cells consisting primarily of CD8^+^ T-cells (30%) along with some NK cells (10%) (Supplementary Fig. 18). There are no significant differences in the CD4:CD8 ratio across clinical groups. Comparative analysis of cellular proportion among RP and NP groups depicted a significantly higher proportion of CD8^+^ exhausted T-cells in the RP samples (*P* = 0.022), whereas NK and CD8^+^ effector T-cells were non-significantly enriched in the NP samples (Fig. 3c, d). The higher enrichment of CD8^+^ exhausted T-cells in the RP samples indicates that RP patients with rapid progression of the disease have a prevalence of exhausted T cells even at the baseline/diagnosis. Pathway analysis was performed to compare the general CD4^+^ and CD8^+^ populations between clinical groups. Pathway analysis on these CD4^+^ T-cells depicted significantly higher enrichment of metabolic pathways such as Hallmark glycolysis and fatty acid metabolism in the patients with rapid progression. In addition, RP CD4^+^ T-cells depicted enrichment for downstream targets of both interferon alpha and gamma (Supplementary Fig. 19a). The pathway analysis on CD8^+^ T subset showed that RP samples were enriched in xenobiotic metabolism, while NP samples were enriched in TNF*α* signaling (Supplementary Fig. 19b). Survival analysis was performed using the MMRF CoMMpass dataset in the Survival Genie tool^19^, and the metabolic pathways which are enriched in rapid progressors, such as, fatty acid metabolism, glycolysis, and xenobiotic metabolism, were found to be associated with poor overall survival (Supplementary Fig. 19c).

We further investigated the gene expression profiles of the three CD8^+^ T-cell subtypes, revealing a range of cells with various degrees of exhaustion or cytotoxic activity. The CD8^+^ memory subset had high expression of markers associated with memory T-cells, such as *KLRB1* and *IL7R*, but had low expression of cytotoxic markers such as *GZMK, GZMB*, or *NKG7*. CD8^+^ exhausted T-cells, in addition to elevated expression of *TIGIT* and multiple genes related to chemokine signaling, such as *CCL3* and *XCL2*, had enriched expression of *GZMK* relative to other T-cell clusters. CD8^+^ effector T-cells show a shift away from *GZMK* and towards *GZMB* and *GZMH*, along with enrichment of cytotoxic and NK cells markers such as *NKG7, GNLY*, and *PRF1* (Fig. 3e). CD8^+^ T-cells from the NP group show enrichment of markers related to the NK cells (*GZMB, GZMA*), whereas the RP group CD8^+^ cells show enrichment of many of the CD8^+^ exhausted T-cell markers, such as chemokine signaling genes *XCL2* and *CCL3L1* (Fig. 3f). The ratio of these more cytotoxic cells to the CD8^+^ exhausted T-cells was significantly enriched in NP group (*P* = 0.048). As aforementioned, the CD8^+^ exhausted T-cell subset shows higher expression of *TIGIT* compared to other CD8^+^ cells (Fig. 3g), along with other exhaustion markers such as *EOMES* and *CD160* which are primarily found in RP samples. Additional CITE-Seq data (ADT) further confirms enriched expression of TIGIT and PD-1 (PDCD1/CD279) in the CD8^+^ exhausted T-cells, and that expression of TIGIT and PD-1 are generally enriched in all RP CD8^+^ T-cell types compared to NP CD8^+^ T-cell types. Overall, the CD8^+^ T-cell compartment shows a shift towards more exhausted effector-memory T-cells in RPs, compared to a more cytotoxic effector phenotype in NPs. Lastly, differential expression between clinical groups within the *GZMB* + CD8^+^ effector T-cells subset showed significant enrichment of multiple genes related to cytotoxicity and T-cell activation in NP samples (*GZMB*, *GNLY*, *TNF*), potentially indicating a better effector function and T-cell activation in NPs (Supplementary Fig. 20, Supplementary Table 2).

### Rapid progressors depicted a higher proportion of alternatively activated M2 macrophages along with activation of complement cascade and lipid processing pathways

**Fig. 4:**
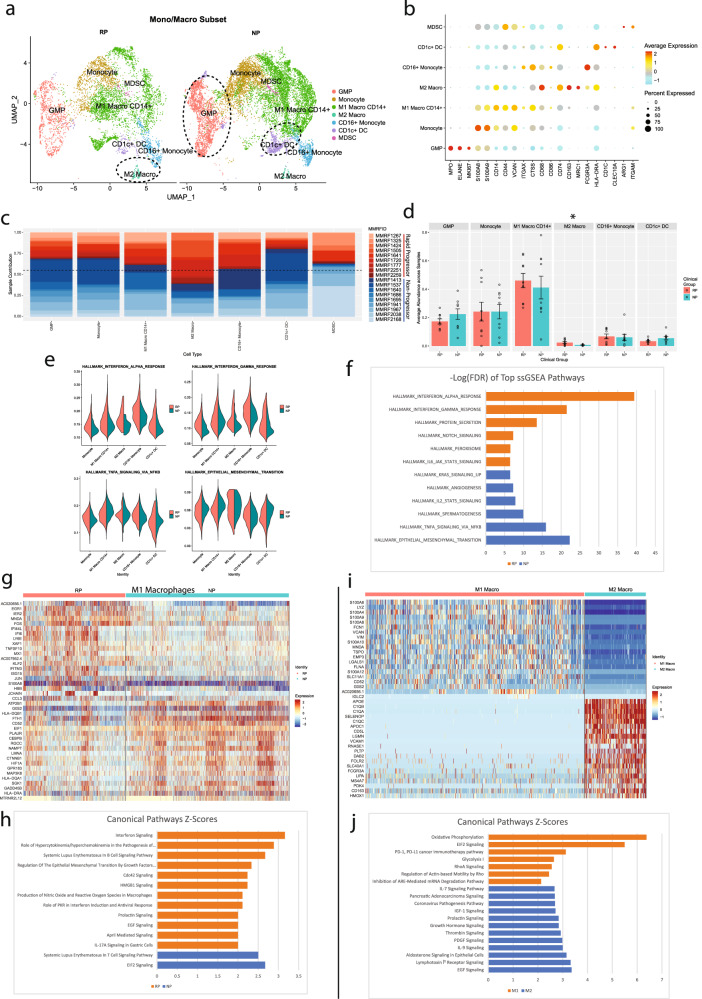
Comparative analysis of the “monocyte and macrophage” and “GMP” immune microenvironment cell subpopulations in multiple myeloma patients with rapid- and no- progression of the disease. **a** A UMAP displaying the monocyte and macrophage subcluster split based on clinical groups (NP and RP). Subclusters were labeled as either Granulocyte-Monocyte Progenitors (GMP), monocyte, CD16 + monocytes, M1 macrophages, M2 macrophage, MDSCs, or CD1c + dendritic cells (DC) based on expression of specific markers. GMP and CD1c + DCs show elevated counts in NP samples. **b** Dot plot demonstrating the key markers for the monocyte and macrophage subtypes. Markers to identify cell types include GMP (*MPO*^+^, *ELANE*^+^, *MKI67*^+^), monocytes (*CD14*^+^, *S100A9*^+^, *S100A12*^+^), M1 macrophages (*CD14*^+^, *CD44*^+^), M2 macrophages (*CD163*^+^, *MRC1*^+^), MDSCs (*HLA-DRA* low, *ITGAM*^+^, *ARG1*^+^), CD16 + monocytes (*CD14*^-^, *FCGR3A*^+^), and CD1c^+^ DCs (*CD1c*^+^). **c** The patient contribution to each cell type cluster indicating most of the clusters consist of cells from multiple patients. The patients from the RP and NP groups are shown with shades of red and blue. Overall, the NP group had a higher proportion of monocytes and macrophages relative to the RP group. **d** Comparative analysis of the myeloid cell types in the RP and NP myeloid subset. Each bar plot depicts the mean proportion of a specific cell type across clinical groups, with error bars displaying standard error of the mean. Individual dots show individual patient samples. M2 macrophages were significantly enriched (*P* = 0.045) within the RP population. **e** Pathway enrichment analysis on the monocyte and macrophage clusters. The Violin plots display the ssGSEA enrichment score of significantly differentially enriched pathways/gene sets between RP and NP groups. The RP group showed significant enrichment of interferon alpha and interferon gamma signaling pathways, while the NP group showed enrichment for TNF signaling and epithelial-mesenchymal transition pathways. **f** A bar graph displaying the top differentially enriched genesets of the monocyte and macrophage clusters based on FDR analysis between NP and RP is also shown. **g** A heatmap, displaying the top differentially expressed markers genes for NP and RP M1 macrophages. Columns represent individual cells, grouped by the RP or NP clinical groups, while rows display individual genes. Relative gene expression is shown in pseudo color, where blue represents downregulation, and red represents upregulation. **h** Selected pathways that are significantly (*P* < 0.01) enriched in the markers differentially expressed in the RP and NP M1 macrophage groups. Each bar represents a pathway with significant activation and inhibition in the RP group based on Z-score calculated using the IPA analysis platform. The pathways that are significantly activated (Z-score > 2) and inhibited (Z-score < −2) in the RP group are shown with orange and blue bars, respectively. **i** A heatmap, displaying the top differentially expressed genes for M1 and M2 macrophages. Columns represent individual cells, grouped by the type of macrophage (i.e., M1, M2), while rows display individual genes. Relative gene expression is shown in pseudo color, where blue represents downregulation, and red represents upregulation. **j** Selected pathways that are significantly (*P* < 0.01) enriched in the markers differentially expressed in the M1 and M2 macrophages. Each bar represents a pathway with significant activation and inhibition in the M1 macrophages group based on Z-score calculated using the IPA analysis platform. The pathways that are significantly activated and inhibited in the M1 macrophages are shown with orange and blue bars, respectively.

To study the alterations in cells of the myeloid lineage, focused analysis was performed after subsetting the myeloid cells. The myeloid subset comprised 16,245 cells of which 10,517 were derived from NP samples and 5728 from RP samples. NP samples show a much higher count of most myeloid cell subtypes compared to RP samples, with a nearly 2:1 ratio of myeloid-lineage cells overall. Based on the correlation of gene expression profiles, myeloid cells clustered into seven different subtypes (Fig. 4a). Cells were annotated as Granulocyte-Macrophage Progenitors (GMP) (*MPO*^*+*^*, ELANE*^*+*^*, MKI67*^*+*^), monocytes (*CD14*^*+*^*, S100A9*^*+*^*, S100A12*^*+*^), M1 macrophages (*CD14*^*+*^*, CD44*^*+*^*, VCAN*^*+*^*, ITGAX*^*+*^*, CD86*^*+*^), M2 macrophages (*CD163*^*+*^*, MRC1*^*+*^), MDSCs (*HLA-DRA* low, *ITGAM*^+^, *ARG1*^+^), CD16^+^ monocytes (*CD14*^*-*^*, FCGR3A*^*+*^), or CD1c^+^ DCs (*CD1c*^+^, *CLEC10A*^+^, MHC-II High) (Fig. 4b). Most of the myeloid cell clusters consist of cells from both NP and RP groups without any patient-specific clusters (Fig. 4c). Comparative analysis of cellular proportion across patient groups reveals significant enrichment of the M2 macrophage cluster in the RPs (*P* = 0.046) (Fig. 4d). However, NPs depicted higher enrichment of immature cell types, such as GMPs, and CD1c^+^ DCs relative to RPs. To explore pathways level dysregulation (if any) among various subtypes of myeloid cells, pathways enrichment analysis was performed. Similar to the T-cell subsets, monocyte, and macrophages from RP samples show enrichment for pathways related to IFNα and IFNγ, whereas NP samples predominantly show enrichment for TNFα via NFkB-related signaling pathways (Fig. 4e, f).

Differential gene expression analysis on M1 Macrophages, the largest myeloid cell subtype, was performed between NP and RP groups. RP samples showed enrichment of multiple genes consistent with enriched interferon signaling, such *as EGR1, IFI6*, and *IFI16*, while M1 Macrophages from NP samples show enrichment of proliferative genes, such as *G0S2* and *CEBPB* (Fig. 4g). Pathway analysis on these DEGs further confirms the enrichment of interferon signaling in RP samples (Fig. 4h).

Differential gene expression between M1 and M2 macrophages was also performed as M2 macrophages are enriched in patients with rapid progression of the disease. M1 macrophages showed enrichment of inflammatory markers, including multiple *S100A* genes, whereas, M2 macrophages were enriched in multiple genes involved in the immune complement cascade (*C1QB, C1QA*), and lipid processing (*APOE*) (Fig. 4i). M2 macrophages also highly expressed *VCAM-1*, which is known to interact with myeloma cells through *VLA-4*^20^. M2 macrophages also showed enrichment of tumor-promoting pathways, such as the platelet-derived growth factor (*PDGF*) signaling^21^ (Fig. 4j).

### Non-Progressors depicted higher enrichment of immature B cell lineages

**Fig. 5:**
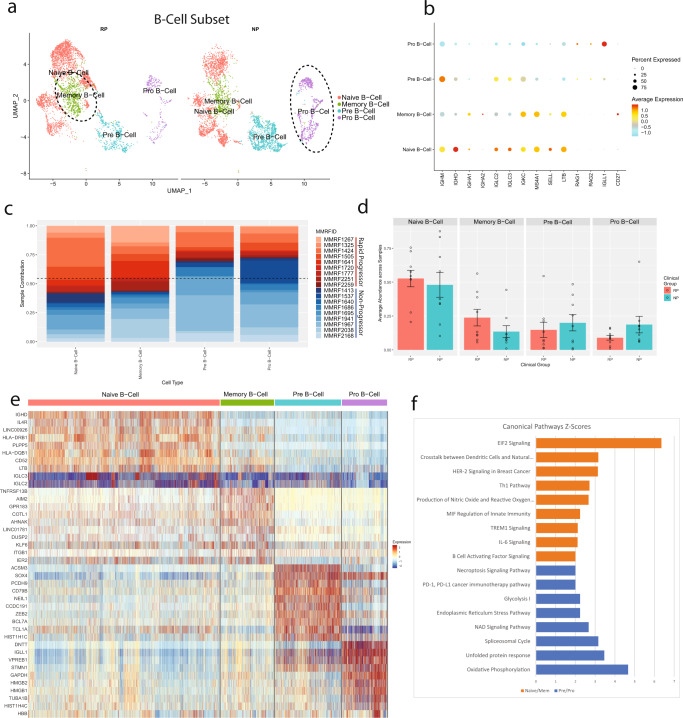
Progenitor and precursor B-Cells are enriched in the multiple myeloma patients with no progression of the disease. **a** A UMAP displaying the B Cell subcluster split based on clinical groups (NP and RP). Subclusters were labeled as either pro B-cell, pre B-cell, memory B-cell, or naive B-cell based on expression of specific markers. **b** A dot plot displaying the key markers used to identify each B cell subtype. Naive B-cells were identified via the expression of *MS4A1*, *SELL*, and *LTB*. Memory B-cells were identified by expression of *CD27*. Pre B-cells were identified by low *MS4A1* expression. Pro B-cells were identified by the expression of *RAG1*, *RAG2*, and *IGL11*. **c** The patient contribution to each cell type indicates most of the clusters consist of cells from multiple patients. The patients from the RP and NP groups are shown with shades of red and blue respectively. The majority of naive and memory B-cells are derived from samples of the RP group, while the majority of pre and progenitor B-cells are derived from samples of the NP group. **d** Comparative analysis of the B-cell types/subtypes in the RP and NP clinical groups. Each bar plot depicts average abundance across patients and error bars show the standard error of the mean. Individual dots represent the abundance of a cell type within an individual sample. Within the B-cell subtypes, there is no significant difference in the average patient ratio. **e** A heatmap, displaying the top differentially expressed marker genes for naive B-cells, memory B-cells, pre B-cells, and pro B-cells. Relative gene expression is shown in pseudo color, where blue represents downregulation, and red represents upregulation. **f** Pathway analysis was performed on the differentially expressed markers between mature and memory B-cell groups versus the pre and pro B-cells. Selected pathways that are significantly (*P*-value < 0.01) enriched in these markers are displayed in the bar chart. Each bar represents a pathway with significant activation and inhibition in naive or memory B-cells based on Z-score calculated using the IPA analysis platform. The pathways that are significantly activated and inhibited in the naive and memory B-cells are shown with orange and blue bars respectively.

To determine dysregulations in B cell repertoire, we performed focused analysis on the clusters enriched with B-Lymphoid lineage cells. This subset consisted of 8009 cells; 4094 derived from NP samples and 3915 derived from RP samples, which formed clusters corresponding to four identified cell types (Fig. 5a). Cells were identified as naive B-cells (*IGHM*^*+*^*, IGHD*^*+*^*, MS4A1*^*+*^), memory B-cells (*MS4A1*^*+*^*, CD27*^*+*^), pre-B-cells (*IGHM*^*+*^*, MS4A1-Low, IGLL1*^*−*^*)*, or progenitor B-cells (*IGLL1*^*+*^*, RAG1*^*+*^*, RAG2*^*+*^) (Fig. 5b). The profile of RP group reflects more mature B cell types (naive, memory), compared to the NP group. On the other hand, NP samples depicted higher enrichment of immature B cells (pre B-cells, pro B-cells) (Fig. 5c, d).

Differential gene expression was performed across the cell types to investigate the differences between the more mature and immature B-cell subtypes. Many of the DEGs are consistent with the standard markers for each category, such as *IGHD* and *IL4R* in the naive B-cells^22^, *AIM2* in memory B-cells^23^, *IRF4* in pre B-cells^24^, and *IGLL1, DNTT*, and *VPREB1* in pro B-cells^25^ (Fig. 5e). In addition, the mature B Cells, show higher expression of MHC-II receptors and receptors involved in BAFF signaling, such as *TNFRSF13B*. Pathway analysis performed on these top markers show that the more mature B cell types are significantly enriched in BAFF and IL6 signaling (*P* < 0.01), both of which are involved in B cell and plasma cell maturation and proliferation (Fig. 5f). The decreases observed in the immature B cell population in the rapid progression group are consistent with previous studies showing the association of decreased progenitor B cell populations with symptomatic or relapsed MM^26–28^.

### Cellular communication predicted dysregulation in BAFF, CCL, and IL16 signaling network

To further investigate potential signaling differences within the immune micro-environment in rapid or non-progressors we performed cellular communication analysis^29^. The potential for cell communication was measured by comparing the average expression of various ligands and receptors among the defined cell types. Using the ligand-receptor expression, an information-flow score is computed for each ligand-receptor pathway between all cell types to indicate communication patterns that are most likely to occur. If two cell types have a high expression of both the ligand and the receptor, then the information flow score between these cell types for this pathway will be higher relative to the other pathways.

**Fig. 6:**
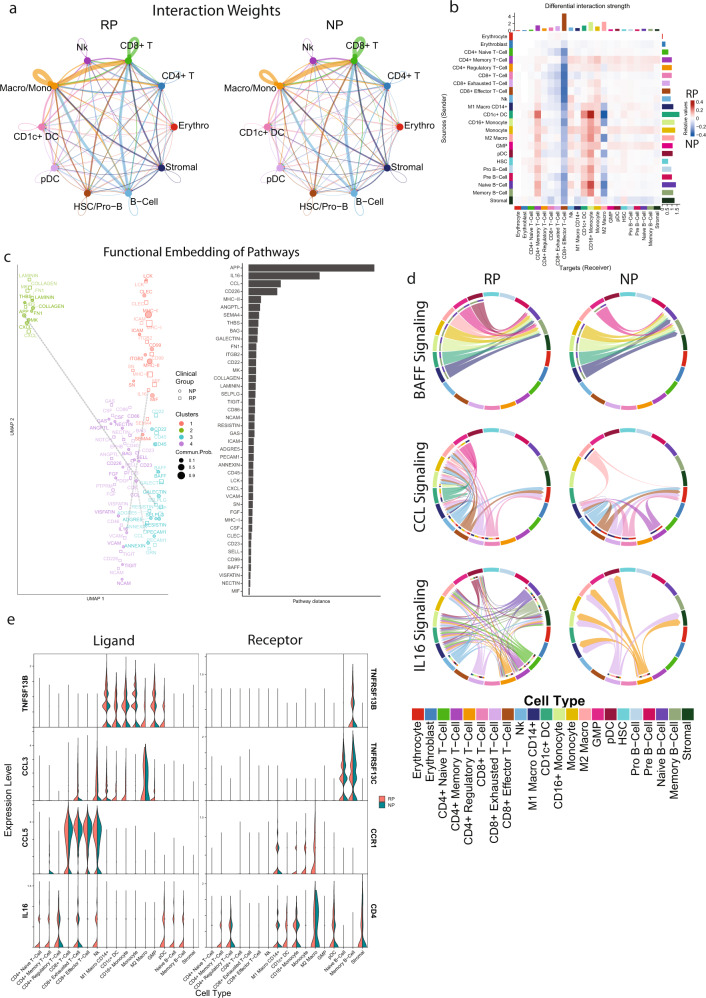
Cell communication analysis reveals enriched signaling pathways and ligand-receptor interactions that are associated with poorer outcome in the rapid progressor group. **a** A circle plot showing the overall communication between cell types in NP and RP groups. The lines in the plot depict the communication among the cell types. Lines are colored by the ‘sender’ cell type, with their thickness corresponding to the relative intensity of cellular communication measured based on ligand and receptor correlation. RP and NP samples showed similar communication patterns between cell types. **b** Heatmap comparing the interaction weights between each cell type in NP and RP groups. Rows correspond to different sender cells, while columns correspond to receivers. The enriched signaling intensity between two cell types in the RP and NP groups are shown in red and blue colors, respectively. Cytotoxic T-cells show enriched received signaling in NP samples from all cell types, while memory and regulatory CD4^+^ T-cells show enriched signaling with myeloid and B-cells in RP samples. **c** Comparison of the signaling structure for individual ligands in NP and RP. On the left, A UMAP embedding of the ligand-receptor pathways was generated based on the similarity of the sender and receiver populations, as defined by CellChat’s functional embedding. Ligands with similar sender and receiver cell types will have similar embeddings. On the right, a bar plot displaying the distance between NP and RP embeddings for each ligand is displayed. Dashed gray lines connect the NP and RP functional embeddings of the top three pathways by pathway distance, APP, IL16, and CCL. **d** Three ligand receptor pairs were isolated for further analysis: BAFF, IL16, and CCL. For each ligand, two chord diagrams are shown indicating the sender and receiver cell types involved in NP and RP. Chords are colored by the sender cell type. BAFF shows a similar signaling structure, with myeloid cells as senders and B-cells as receivers. CCL signaling involves T-cells as senders and myeloid cells as receivers in NP and RP samples, though RP shows additional myeloid cell types as receivers, along with some CCL secretion by myeloid cells. IL16 shows large structural differences, in which both groups have CD8^+^ exhausted and CD4^+^ regulatory T-cells as senders and myeloid cells as receivers, but RP samples show additional expression by B-cells and other CD4^+^ T-cells. **e** Violin plot comparing the expression of the ligands and receptors involved in BAFF, IL16, and CCL between NP and RP samples across all cell types.

First, the overall signaling structure was compared between the NP and RP samples. In both NP and RP, the CD8^+^ T-cells are the primary receivers of cell–cell signaling, and B-cells, monocytes, and macrophages act as primary senders (Fig. 6a). The overall communication structure is similar, and there are no cell types where ligand-receptor interactions are exclusively restricted to one cell population. CD8^+^ effector T-cells show increased signaling received in the NP group relative to the RP group from all cell types, while CD4^+^ memory T-cells, CD1c^+^ DCs, and CD16^+^ monocytes show higher signaling received in RP samples from other myeloid or B-lymphoid cells (Fig. 6b). These differences seem to be primarily driven by MHC-II–CD8a interactions in NP CD8^+^ effector T-cells, and MHC-I–CD4 interactions in CD4^+^ RP cells (Supplementary Fig. 21).

We also compared the signaling structure and expression of individual ligand-receptor interactions in non-progressors and rapid progressors. First, to compare the signaling structure, ligands were embedded based on the sender and receiver cell types involved in their signaling pathway. Ligands that share closely related sender or receiver cell types will have similar embeddings. These embeddings resulted in four clusters, with cluster 2 consisting of ligands associated predominantly with the stromal cell population, and clusters 1, 3, and 4 with the immune cell population. To identify pathways with large differences in structures, the embedding distance between each pathway in NP and RP was computed (Fig. 6c). *APP*, *IL16*, and *CCL* were identified as ligands with the largest differences in their structural embedding. Three signaling pathways were selected for further investigation based on both the signaling structure (Fig. 6d) and differential gene expression (Fig. 6e) between NP and RP samples: BAFF, CCL, and IL16.

The BAFF signaling pathway overall has a similar structure in NP and RP, with myeloid cells being the primary secretors of the ligand *TNFSF13B*, and the memory and naive B-cells populations being the primary receivers through receptors *TNFRSF13B* and *TNFRSF13C*. Elevated serum levels of *TNFSF13B* have been noted previously in MM patients^30^, and BAFF signaling can have pro-proliferative effects on myeloma cell lines^31,32^. Interestingly, our analysis reveals that although the receptors on B Cells, *TNFRSF13B* and *TNFRSF13C*, are similarly observed in both groups of samples, the RP samples show consistent enrichment of the ligand, *TNFSF13B*, in multiple myeloid cell types compared to NP samples. Survival analysis using survival genie tool^19^ indicates that disproportionately elevated expression of the ligand (TNFSF13B) relative to the B cell receptor (TNFRSF13B) is significantly associated with poorer overall survival across the MMRF CoMMpass dataset (*P* = 0.0067) (Supplementary Fig. 22).

The CCL signaling pathway involves the secretion of *CCL3* and *CCL5* from CD8^+^ T-cells and some myeloid cells and the expression of the receptor *CCR1* on various myeloid cells. Previously, we noted that the CD8^+^ exhausted T-cells, primarily found in RP samples, were enriched for multiple chemokine-related genes, including *XCL2*, *CCL3L1*, and *CCL4L2*. Here, we see additional receivers and senders in RP samples compared to NP samples, primarily in the myeloid subset. DCs, monocytes, and M2 macrophages express higher levels of the receptor *CCR1* in RP samples, whereas prior *CCR1* was primarily elevated in M1 macrophages. In addition, we observed the expression of *CCL3* in RP monocytes, DCs, and macrophages. The ligand *CCL3* specifically has a pro-survival and pro-proliferative effect on myeloma cell lines^33^, and can disrupt the natural progenitor population^34,35^. Elevated expression of *CCL3*, *CCL5*, and *CCR1* is significantly associated with poorer outcomes in the MMRF CoMMpass dataset (*P* = 0.001) (Supplementary Fig. 22).

Lastly, IL16 shows large structural differences in the signaling pathway between NP and RP samples. IL16 involves secretion from CD8^+^ exhausted T-cells and CD4^+^ regulatory T-cells in both NP and RP samples and binds to CD4 on myeloid and T-lymphoid cells. RP samples exclusively show IL16 secretion from B cells, and show additional expression in NK cells, CD4^+^ memory T-cells, and CD4^+^ naive T-cells. Previous studies have shown IL16 elevation in MM marrow samples, and pro-proliferative effects on myeloma cell lines^36^. However, within the myeloma cell compartment, overexpression of IL16 relative to the CD4 receptor is associated with slightly better outcomes in the MMRF CoMMpass dataset (*P* = 0.038) (Supplementary Fig. 22).

## Discussion

The long-term outlook for patients with advanced MM remains dismal. The therapeutics for MM have undergone a paradigm shift in the last decade with the advent of immune therapy, resulting in sustained disease responses in a subset of previously incurable patients. Immune therapy recruits multiple effectors that target mutated tumor antigens, develop lasting memory responses, and overcome mechanisms of therapeutic resistance. Clinical trials for immunotherapies across multiple cancers have established that this anti-tumor immune response is a key determinant of positive treatment outcome^37^. Even though targeted and antibody-mediated therapies have improved progression-free survival in MM patients, many patients still develop resistance or eventually relapse, due to the existence of a small number of therapy-resistant tumor stem cells or minimal residual disease^38^. It is imperative to develop cancer therapies that disrupt tumor-associated tolerance, activate, and selectively expand tumor-specific lymphocytes, and at the same time maintain immune-regulatory protection. Therefore, it is key to study the tumor microenvironment in-depth to decipher its role in achieving long-term remission. In the current era of single-cell profiling, studying the contribution of individual tumor microenvironment cells in a high-throughput manner is feasible. In the last 5 years, ~2000 single-cell studies have been conducted to study tumor cells along with microenvironment. A recent study from Zhang et al.^10^ investigating the temporal progression of MM from the precursor stage, revealed the role of a compromised immune microenvironment, specifically T-cells, in the progression and poor outcome of MM. The major limitation of single-cell approaches is the requirement of viably frozen or fresh patient samples for performing high-quality cellular profiling. In this study, we optimized experimental protocols for thawing cells and analytical workflows after implementing multiple batch correction approaches to generate high-quality single-cell profiling data from viably frozen clinical bone marrow samples.

The first question that we attempted to answer in this pilot study was on technical variations in the single-cell profiling data obtained from geographically different processing centers. We performed single-cell profiling on samples from the same set of patients at three medical centers across the United States (BIDMC in Boston, WashU in St. Louis, and MSSM in NYC). All major cell populations are consistently identified in samples processed across three centers, and marker genes for these cell types have consistent expression across centers. The relative cellular abundance for each sample showed subtle differences in samples processed at BIDMC, which performed CITE-Seq, compared to those processed at MSSM and WashU, which only performed scRNA-seq. These differences might be due to additional processing time for CITE-Seq antibody labeling impacting the viability of these samples. These variations in cell abundance can be mitigated in future studies by utilizing the same protocol to generate single-cell profiling data across various processing centers (including with incorporation of dead cell removal steps to ensure high viability of single-cell preps). A previous study comparing scRNA-seq data derived from fresh and frozen samples of the same patient identified a similar pattern, where gene expression between fresh and frozen samples was consistent, but the relative cell abundance changed, with frozen samples tending to have higher mitochondrial transcripts^39^. This study demonstrates the feasibility of integrating single-cell profiling data generated in multi-center national and international clinical trials with minimal technical variations.

Further comparative analysis of the cellular abundance of different cell types depicted significant alterations in cellular abundance and the transcriptome profile of cells from T-cell and myeloid lineages between RPs and NPs of multiple myeloma. In the T-cell lineage, significant alterations were identified within the CD8^+^ T-cell population resulting in impaired effector functions in the T-cells of RPs. Rapid progressor samples contain a higher number of exhausted CD8^+^ T-cells compared to other T-cells. The subset of CD8^+^ T-cells expressing these exhaustion markers (*TIGIT*, *EOMES*, *CD160*, PD-1) corresponds with high *GZMK* expression and multiple chemokines signaling markers (*CCL3*, *CCL4*, *XCL2*, *CMC1*). This is in contrast with NP samples, whose CD8^+^ T-cells showed high expression of *GZMB* and cytolytic markers (*PRF1*, *GNLY*). A similar exhaustive GZMK^+^ phenotype has recently been described as a pre-dysfunctional exhaustive progenitor T-cells, distinguished by high surface expression of PD-1 and TIGIT along with the expression of effector-memory like marker^40,41^. This exhausted GZMK^+^ cell type has also been described in other studies as an inflammatory aging-associated T-cells, as their abundance shows a correlation with the age^42^. These T-cells are associated with reduced proliferation and effector functions in response to stimulation, which ultimately would impair anti-tumor immune response. Exhausted T-cells with the enriched expression of CTLA-4, PD-1, CD160, and 2B4 have been previously linked with poor outcomes in MM patients^16,43^.

In addition, we observed significant enrichment of *GZMB*^+^ CD8^+^ effector T-cells in NP patients relative to *GZMK*^*+*^ CD8^+^ exhausted T-cells. Cytotoxic T-cells normally play a critical role in anti-cancer immune responses. Previous studies have noted that, relative to healthy controls, MM patients typically show a depletion of the CD8^+^ T memory subset, with enrichment of the effector T-cell population^2,16^. However, studies comparing patients with long-term disease remission following autologous stem cell transplantation observed to have a higher abundance of the cytotoxic CD8^+^ T-cell and NK subsets as compared to symptomatic MM, MGUS, and healthy controls^26^. It is possible that the enrichment of the effector T-cell subset is required for anti-tumor control, and patients with an enriched exhausted effector memory subset, fail to clonally expand a functional effector T-cell population. Within the effector GZMB^+^ subset, we do see significant enrichment of effector and signaling genes, such as *GNLY, GZMB*, and *TNF* in the NP samples, potentially indicating improved effector function (Supplementary Table 2, Supplementary Fig. 20).

Other immune cell populations, such as the monocytes and macrophages, also depicted dysregulation between RP and NP patients. The myeloid subset shows a significant enrichment of M2 macrophages in rapid progressors. M2 macrophages were identified based on the expression of *CD163*, and are typically considered to promote tumor growth and survival, compared to the more inflammatory M1 macrophage phenotype^44^. A previous study investigating the role of tumor-associated macrophages in MM identified that patients with a higher M2 macrophages involvement showed both poor response to dexamethasone, and lower progression-free and overall survival compared to those with a higher M1 macrophage contribution^45^. In addition, M2 macrophages in MM bone marrow demonstrated high *VCAM-1* expression relative to other myeloid cell types. Myeloma cells are known to adhere to bone marrow stromal cells through VCAM-1–VLA4 interaction, which can activate pro-proliferative signaling pathways in the myeloma cell lines^20^.

In addition to dysregulation of certain cell populations between RPs and NPs, we also observed alterations in specific signaling pathways across the immune cells, including enriched signaling of BAFF, CCL, and IL16 from myeloid cells, T and myeloid cells, and B cells, respectively. Dysregulation of these signaling pathways have been previously associated with MM progression. For example, BAFF/TNFSF13B, APRIL, and TNFSF13C, bind to TNFRSF13B, TNFRSF17C, and TNFRSF13C receptors, found on mature B-cells, memory B-cells, and plasma cells, respectively. Multiple studies have correlated elevated serum levels of BAFF with cancer progression^31,46^. Though BAFF’s highest binding affinity is with the B-cell receptor variants, *TNFRSF13B/C*, there have been reported in vivo cases where myeloma cells aberrantly express these receptors in addition to the normal plasma cell variant, TNFRSF17, further increasing the sensitivity of myeloma cells to BAFF secretion^47^. Interaction of these ligands with the corresponding receptors on myeloma cells can activate the *NFkB* pathway, ultimately aiding myeloma cell survival through the enrichment of anti-apoptotic markers such as MCL-1^32,48^. Though it is possible for mutations in the malignant plasma cells to activate the *NFkB* pathway independent of signaling from the bone marrow, this is an event that typically occurs in advanced stages of disease progression, indicating that external activation of *NFkB* through signaling pathways such as BAFF could play a critical role in the therapeutic resistance of malignant plasma cells during the early phases of disease^49^. The therapeutic Ataciept^47^, which specifically targets the BAFF/APRIL ligands secreted by the BME, has been explored in MM treatment. A phase I study of Atacicept showed stabilization of the disease following treatment^50^, though targeting the ligand directly does increase the risk of secondary infection through the depletion of normal mature B cells. Overall, this supports the critical role these signaling pathways can play in the progression of the disease. Other signaling molecules notably enriched in fast-progressors, such as *CCL3* and *IL16*, can be associated with pro-proliferative effects on malignant plasma cells^33,36^, impaired progenitor differentiation in the bone marrow^34^, and anemia due to disruption of normal erythropoiesis in the bone marrow^35^^.^. We hypothesize that these enriched signaling pathways in the immune compartment may contribute to a favorable BME for myeloma cells in the rapid progressor population. Therapies directly targeting these pathways could improve the outcome in these non-responsive rapidly progressing patients.

This pilot study lays the cornerstone for the development of a MM single-cell immune atlas that will include treatment-naive (baseline or diagnosis) as well as post-therapy samples to enlighten the role of the tumor microenvironment in multiple myeloma. This multi-center study demonstrated that gene expression and key cell markers in scRNA-seq data are similar across different centers, allowing us to obtain and process more bone marrow biopsy samples at multiple centers instead of restricting to a single medical center. Integrating single-cell profiling data from multi-center national and international trials will enable the identification of novel potent biomarker(s) for disease diagnosis and therapeutic interventions critical for a better prognosis for MM patients. Furthermore, the pilot study identified the enrichment of exhausted T-cells with impaired effector functions and M2 macrophage as potential contributing factors in rapid progressing MM samples collected at the time of disease diagnosis. These findings will be further validated in an expanded cohort in collaboration with Multiple Myeloma Research Foundation immune atlas consortium. The analysis on baseline samples will assist in identifying the high-risk patients to plan a personalized follow-up and treatment approach. The identified association of T-cells and macrophages phenotypes with clinical outcome opens avenues for developing novel biomarkers for monitoring therapeutic response. In addition to samples collected at the time of disease diagnosis, single-cell profiling of bone marrow biopsies obtained at relapse and remission will be used to further expand these findings.

## Methods

### Ethics approval and participant consent

All samples involved were obtained from the MMRF CoMMpass clinical trial (NCT01454297). Procedures involving human participants as part of this trial were performed in accordance with the ethical standards of the MMRF research committee. Written informed consent was obtained from all patients for the collection and analysis of their samples and clinical information by the MMRF. The Institutional Review Board at each participating medical center approved the study protocol. The list of all participating institutes which have approved the study protocol is viewable under the ClinicalTrials.gov identifier NCT01454297.

### Experimental model and human subject details

Newly diagnosed MM patients (*n* = 18) from the MMRF CoMMpass study (NCT01454297), comprising both RPs (*n* = 9) and NPs (*n* = 9) were included in the study (Supplementary Table 1). Demographic, clinical, and genetic information about these patients is also available in Supplementary Table 1. Forty-eight aliquots of viably frozen CD138^-^ BM samples (acquired at diagnosis prior to any treatment) from these 18 patients were processed at three medical centers/universities (Beth Israel Deaconess Medical Center (BIDMC), Boston, Washington University (WASHU) in St. Louis, and Mount Sinai School of Medicine (MSSM), NYC) with technical replicates of select patients. BIDMC processed 20 samples from 18 patients, MSSM processed 7 samples from 7 patients, and WashU processed 21 samples from 17 patients. BIDMC captured both cell surface proteins (*n* = 29) and gene expression of single cells via CITE-Seq (Supplementary Table 3) while WashU and MSSM processed samples for only gene expression of single cells.

### Method details

#### CD138^−^ cells isolation and cryopreservation of cell samples

Bone marrow aspirates from the Multiple Myeloma Research Consortium (MMRC) tissue bank were separated into CD138^+^ (myeloma cells) and CD138^−^ (immune, bone marrow cells) fractions using immunomagnetic cells selection targeting CD138 surface expression (automated RoboSep and manual EasySep from StemCell Technologies Inc.). Following magnetic separation, the CD138^-^ cells fractions were viably frozen. Briefly, the CD138^-^ cells were centrifuged at 400 × *g* for 5 min. The resulting cell pellet was resuspended in freezing media consisting of 90% FCS and 10% DSMO at a concentration of 5–30 million cells per ml. Cell concentrations and vial locations were documented, before being stored in liquid nitrogen for future use.

### Processing frozen single-cell suspensions for RNA sequencing

Single-Cell RNA sequencing (scRNA-seq) was performed on viably thawed BM samples using a droplet-based high throughput system (10x Genomics Inc.), which captures single-cells along with uniquely barcoded primer beads together in tiny oil droplets enabling large-scale parallel single-cell transcriptome studies. Briefly, the frozen MM samples were thawed rapidly at 37 ^o^C, serially diluted in warm media, followed by centrifugation at 400 × *g* for five minutes to pellet cells (viability after thawing ranged from 75 to 100%). The pelleted cells were resuspended one more time in warm media to wash off the remaining freezing media/dead cells. For 10x Genomics compatible single-cell suspensions, the final washed cell pellets were resuspended in PBS + 0.04%BSA, before proceeding with the scRNA-seq workflow (WashU, MSSM) or CITE-Seq workflow (BIDMC). The single-cell suspensions (verified microscopically for the absence of clumps) mixed with gel beads and reverse transcription (RT) mix were processed using 10x Genomics workflow to generate digitally barcoded stable and uniform single-cell droplets (gel bead-in-emulsions; GEMs) in the Chromium Controller (10x Genomics, PN: 110211). Following RT and cDNA amplification, the scRNA-seq libraries were prepared using the Chromium Single cell 3’ Reagent kits v3 (10x Genomics, PN: 1000075). Massive parallel sequencing was performed on the scRNA-seq libraries using the Novaseq S4 (Illumina Inc.) platform. We aimed to capture the expression of 8000 cells per sample with ~50,000–100,000 reads per cell. We captured a median of 3628 cells per sample prior to filtering with ~1000–2000 genes per cell.

### Single-cell surface protein expression and gene expression assay

Cellular indexing of transcriptomes and epitopes by sequencing (CITE-Seq) using TotalSeq B antibody-oligo conjugates (Biolegend) along with 10x Genomics 3’ reagent kits enable simultaneous detection of proteins and mRNA in single-cells. TotalSeq B antibodies along with 10x Genomics 3’GEM library and gel bead kit v3 (PN: 1000075) and feature barcode library kit (PN: 1000079) were used to process samples at BIDMC, Boston to simultaneously capture select cell surface proteins expression and gene expression. A panel of 29 select antibody-oligo conjugates or antibody-derived tags (ADT), along with three IgG isotype control ADTs, were used to capture surface marker expression (Supplementary Table 3). Briefly, viably thawed single-cell suspensions (as explained in the above section for processing single-cells) were labeled with the ADTs, washed to remove unbound antibodies, and used to generate ADT-labeled single-cell GEMs according to 10x Genomics protocol. Following RT and cDNA amplification, the two types of cDNA (ADT and GEX) were separated using SPRIselect beads (Beckman Coulter, cat. no. 23318), which were then used to generate ADT and GEX libraries, that were pooled and sequenced according to manufacturer’s protocol.

### Analysis of single-cell RNA sequencing data

Demultiplexed fastQ files from each sample were aligned using *cellranger count*^51^ against a reference human genome (hg38). Count matrices of all samples processed at each center were then combined and normalized using the cellranger aggr pipeline to create a per-center feature matrix. Low quality cells were filtered using Seurat^52^ to keep only cells with >200 unique genes, >500 UMI reads, and <30% mitochondrial UMIs. Potential doublets were also removed by keeping only cells with <10,000 UMI reads. In addition, to determine the impact of more stringent mitochondrial cutoff on cellular clustering pattern and abundance, we also performed the analysis with 20% mitochondrial cutoff (Supplementary Figs. 2–5). The feature matrices were normalized using the *SCTransform* algorithm, regressing out the per cell UMI count, the number of unique features per cell, and the percent mitochondrial reads mapped to a cell. To correct for any batch effect the samples count matrices from three centers were normalized and integrated using integration anchors-based batch correction approach using the Seurat package. The cells in the resulting integrated assay were visualized using a Uniform Manifold Approximation and Projection (UMAP) embedding. Similar cells were clustered together via Louvain clustering on the top principal components of the integrated assay using Seurat package^52^.

### Subcluster analysis and cell labeling

Clusters were first manually labeled as “erythrocytes”, “erythroblasts”, “T-cells”, “Cyto-T”, “NK/T”, “monocyte/macrophage”, “GMP”, “B-Cells”, “HSC”, and “Plasma/Myeloma cells” based on cell-specific canonical marker expression (Supplementary Table 4). Cells identified as “T-cells”, “monocytes/macrophages”, or “B-Cells” were isolated, and the integration process for these cells were repeated for detailed analysis within these specific cell types. Plasma cells were identified based on the plasma cell-specific gene markers including *JCHAIN, MZB1*, and *SDC1* (Supplementary Fig. 1a, b). We further evaluated the heterogeneity of plasma cell clusters by analyzing the composition of different clusters. To ascertain the malignant phenotype of the plasma cells we also performed copy number variation (CNV) analysis described in the next section. The malignant plasma cells were removed from the subsequent analysis to determine the impact of the non-malignant BME on clinical outcomes.

Clusters were manually labeled as various cell types (M1 macrophage, CD8^+^ exhausted T-cell, etc.) based on the differentially expressed gene markers for a given cluster (Supplementary Table 4). For some clusters, surface marker expression from samples processed at BIDMC was used to further confirm cell type (CD4 expression for CD4^+^ T-cells, CD45RA and CD45RO for naive and memory T-cells respectively), and to verify the presence of T-cell exhaustion markers (TIGIT, PD-1). Refined cell labels, identified during subcluster analysis, were transferred back to the full object for analysis involving all cell types. In situations where cluster identity was ambiguous, individual clusters were subclustered further at a higher resolution until specific cell subtypes could be assigned. Markers to identify cell types were derived from a variety of sources. A supplementary table with the markers used for annotation is available (Supplementary Table 4)^25,53–59^.

### Single-cell copy number variation analysis

The malignant phenotype of the MM plasma cells is associated with somatic copy number alterations resulting in gene amplifications and gene deletions. In this study, we used InferCNV algorithm^17,60^ to predict the CNVs in the plasma cells identified based on the expression of specific markers. The algorithm determines dysregulations of genes across chromosomal positions in the tumor cells and normal cells to identify regions in chromosomes of tumor cells that are over- or less-abundant as compared to normal cells. We performed CNV analysis comparing plasma cells with either mature B cells from this study or normal plasma cells from the human single-cell atlas initiative^18^. Gene expression intensities are represented in a heatmap where genes in the scRNA-seq dataset were sorted by genomic position and were further ordered within each chromosome.

### Correlation of cell types across centers

To determine the correlation of the cell types across different centers, we used the Clustermap tool^61^. The final cell type labels from the integrated object were transferred to the un-integrated Seurat objects for each center. For each center, the top differentially expressed markers were computed for each cell type relative to all other cells from a given center. These were computed with Seurat’s “FindAllMarkers” command filtering genes expressed in <25% of cells of a given type, a minimum log-fold change of 0.25, and a maximum *P* value of 0.01. These markers were correlated with the top markers for all other cell types from all other centers to ensure that the cell type definitions were consistent across all three centers.

### CITE-Seq data normalization/processing

The raw read counts for the ADT analytes that included 29 protein targets, and three isotype controls (Supplementary Table 3) were generated using the cell ranger package from 10x Genomics. The preliminary analysis and quality control depicted non-specific signals for the multiple ADTs. To reduce the noise due to non-specific antibody binding, the background signal was subtracted based on the signal of control isotype antibodies. The gene expression data were preprocessed to remove outlier cell types. The background-subtracted ADT data was normalized using the “CLR” method in the Seurat Package and scaled before integrating the normalized gene expression data from each sample.

### Analysis of CITE-Seq with scRNA-seq data

After clustering and labeling was performed on scRNA-seq data, cells with corresponding surface marker data were subset and RNA and Surface Marker assays were merged. Surface marker expression was used to confirm and refine cell labels for the previously identified clusters. Differential expression of surface protein markers was performed using the Wilcoxon signed-rank test to identify clusters or clinical groups which are enriched for specific surface markers.

### Pathways and systems biology analysis

Pathways and systems biology analysis was performed using the Ingenuity Pathway Analysis (IPA) software package (Qiagen)^62^. The differentially expressed genes (DEGs) for different cell types or phenotypes (i.e., M1 vs M2 macrophages, RP vs NP M1 macrophages, and Immature vs Mature B-Cells) were obtained based on an absolute log-fold change ≥0.25, >10% of cells expressing gene, and *P* < 0.01 based on Wilcoxon signed-rank test. These DEGs were used to identify significantly affected pathways using the IPA platform. The knowledge base of IPA platform consists of functions, pathways, and network models derived by systematically exploring the peer-reviewed scientific literature. A detailed description of IPA analysis is available at the Ingenuity Systems’ website (http://www.ingenuity.com). It calculates the multiple test corrected *P* value for each pathway according to the fit of user’s data to the IPA database using one-tailed Fisher exact test. The pathways with raw *P* < 0.01 were considered significantly affected. In addition, IPA also calculates Z score indicating directional effects on pathways with the Z-score >2 was defined as the threshold of significant activation, while Z-score < −2 was defined as the threshold of significant inhibition.

### Gene set enrichment analysis

Single-cell gene set enrichment analysis (ssGSEA) was performed using the escape R package^63^. The package was modified to operate on the SCT normalized data and to assume a gaussian distribution for the expression data. ssGSEA scores were computed for each cell using the Hallmark gene sets acquired from MSigDB database^64^. A Welch *T*-test was performed on the distribution of ssGSEA scores using escapeR’s getSignificance function to identify significantly enriched pathways between clinical groups within specific cell subsets. The gene sets with multiple tests corrected *P* < 0.05 were considered significantly different.

### Survival analysis

All survival analysis was performed using the Survival Genie platform^19^ with the MMRF CoMMpass dataset (dbGaP Accession phs000748.v7.p4). This dataset includes bulk RNA-seq data from the CD138^+^ fraction derived from MM patients. Survival analysis was performed using the gene set option across primary MM samples. Survival analysis on the ssGSEA pathways was performed using all genes included in the corresponding Hallmark gene set. Survival analyses for cell communication ligands and receptors data were performed using gene set (if both ligand and receptor were overexpressed) or gene ratio if only the ligand is overexpressed. Tumor samples were categorized into high and low gene expression groups using an optimal cut point (cutp) estimated based on martingale residuals^65^ of the GSEA score for determining association with overall survival. The results were considered significant if the *P* values from the log-rank *t*-test < 0.05.

### Cellular communication and interaction analysis

Cellular communication analysis was performed using the CellChat software platform^29^. Cells from each clinical group were isolated, and ligand-receptor (L-R) analysis was performed on the clinical groups independently using the standard CellChat analysis workflow. Information flow scores for each signaling pathway provided by the CellChat L-R interaction database were computed between all previously defined cell types. Information flow characterizes the likelihood of cell–cell interaction occurring through a given pathway. Cells with high expression of a known ligand will have high information flow scores with cells that have high expression of the matching receptor. The resulting CellChat objects on the RP and NP clinical groups were merged to compare information flow scores for specific cell types and pathways. To compare the overall signaling structure between cells in NP and RP samples, interaction weights were used, which sum the information flow of all L-R interactions between two cell types. To compare the signaling structure of individual L-R interactions, we first used CellChat’s network embedding feature to identify pathways with large differences in the sources or targets between RP and NP clinical groups. We used a functional embedding, which creates a two-dimensional embedding of all L-R interactions based on the gene expression of the source and target cells involved in an individual L-R network. The Euclidean distance between the same L-R pathway in both NP and RP clinical groups was used to identify pathways with large differences in signaling structure. Select pathways were highlighted based on the differential expression of the given genes between clinical groups, degree of signaling structural differences, and potential clinical relevance.

### Quantification and statistical analysis

All statistical analysis was performed with R. Cellular abundance across samples is displayed as the mean ± standard error. Significance for cell ratios is computed with Welch’s *T*-test without multiple test corrections. The significance of differentially expressed genes is determined based on the Wilcoxon rank test with Bonferroni multiple test correction^66^. Significance for ssGSEA pathways is computed with escape’s getSignificance function, internally using multiple tests corrected *P* value estimated using Welch’s *T*-Test with Benjamini-Hochberg multiple test correction. Heatmaps of gene expression were made with DoHeatmap function in the Seurat package. Volcano Plots based on differentially expressed genes were made using the EnhancedVolcano R package. Violin plots to compare expression across cell types or clinical groups were made using VlnPlot function in R. Circos and Chord diagrams are rendered using the Circlize R package^67^. Shannon’s entropy was computed per cell using 100 neighbors and 20 principal components using the CellMixS R package^68^ for assessing the impact of different processing centers on cellular expression profiles.

## Supplementary information

Reporting Checklist

Supplemental Materials

## Data Availability

A dataset containing the scRNA-seq and CITE-Seq data used in this analysis is available at the NCBI Bioproject (PRJNA765009) from the Immune Atlas Consortium. Access to additional metadata and sample information for those enrolled in the Multiple Myeloma Research Foundation (MMRF) CoMMpass study (NCT01454297) can be requested from the MMRF at https://research.themmrf.org/. For more information on the MMRF CoMMpass study, see the corresponding clinical trial, NCT01454297 and the MMRF Researcher Gateway (https://research.themmrf.org/).

## Code availability

All the code used for data analysis and generation of figures will be available upon request. Software used includes Cellranger 3.1, Seurat v4 (R Package), escape (R Package), CellChat (R Package), Circilize (R Package), ClusterMap (R Package), CellMixS (R Package), and Ingenuity Pathway Analysis.
