# Supplementary material for: Cross center single-cell RNA sequencing study of the immune microenvironment in rapid progressing multiple myeloma
Source: NPJ Genom Med. 2023 Jan 26;8:3. doi: 10.1038/s41525-022-00340-x (PMC9879959; doi:10.1038/s41525-022-00340-x)
Supplement: Supplementary file 2 — Supplemental Materials [file 41525_2022_340_MOESM2_ESM.pdf]

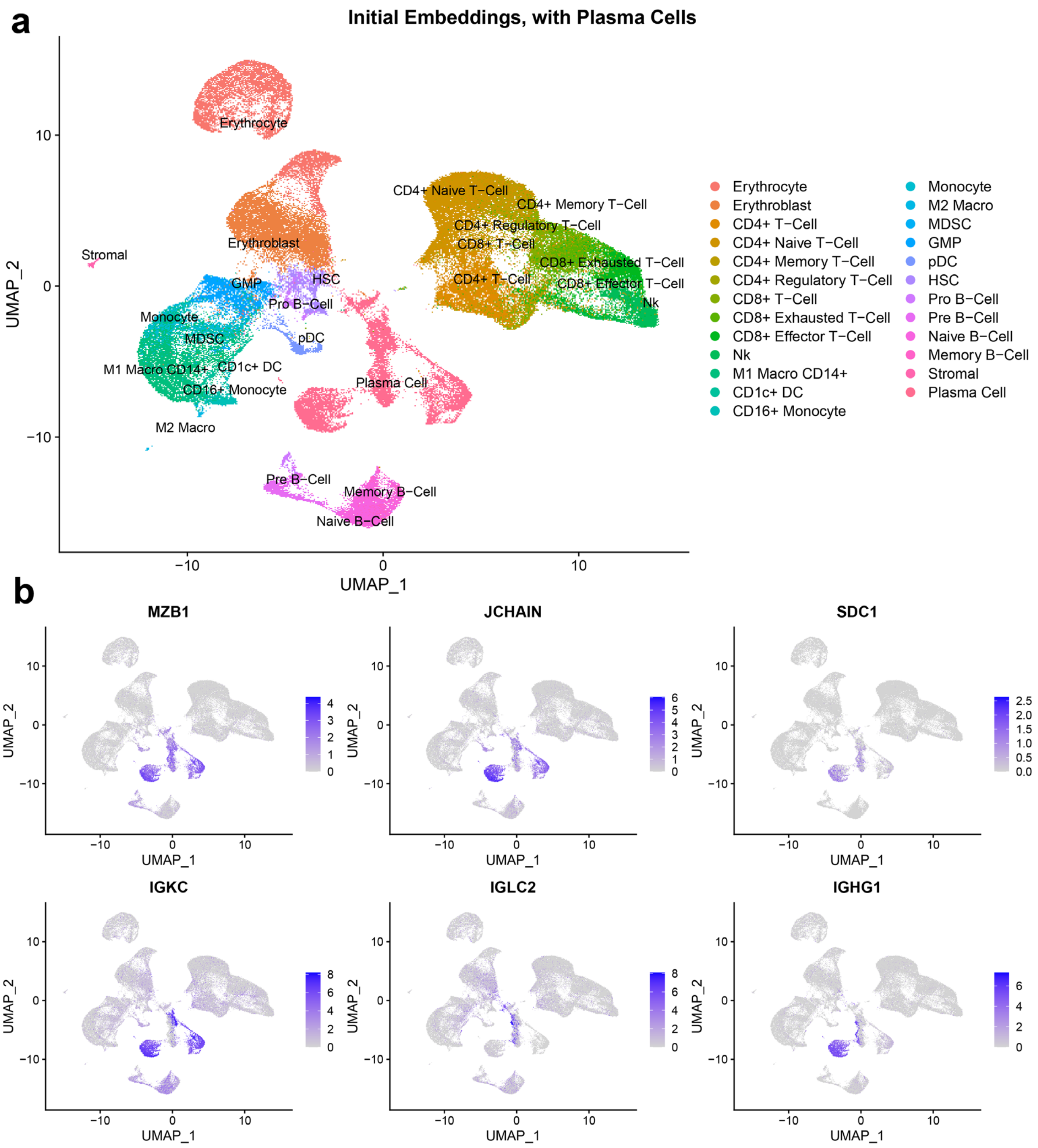

Supplementary Figure 1: Analysis of the Plasma cells identified in the study. a. UMAP embeddings of bone marrow microenvironment cells along with Plasma cells identified in the study. **b.** Feature plots of genes to identify the plasma cell populations. Plasma cells were identified via a variety of markers, including *JCHAIN, MZB1, SDC1*, and high expression of a light or heavy chain immunoglobulin.

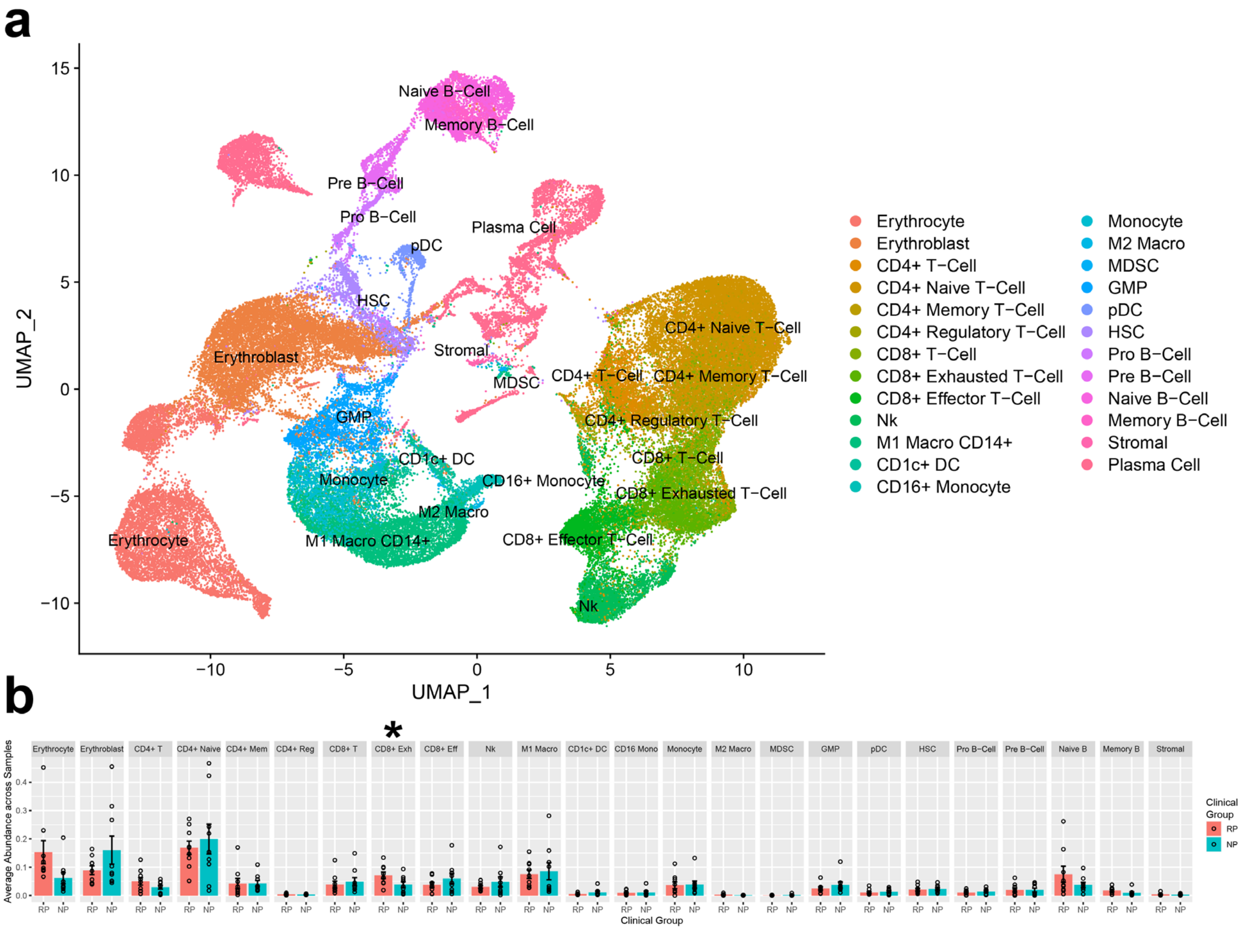

Supplementary Figure 2: Analysis of single-cell data using lower mitochondrial cutoff. a. UMAP of samples from all centers filtered using a 20% mitochondrial UMI cutoff to understand the impact on clustering patterns. **b.** Comparison of the average ratio of the various non-plasma cells in RP and NP patient samples. RP samples have a significantly (P = 0.049) higher ratio of $CD8^{+}$ Exhausted T-cells relative to the rest of the $CD138^{-}$ population. Each bar plot depicts the mean proportion of cells within individual samples with an error bar displaying the standard error of mean. Dots represent the proportion of a given cell type in an individual sample.

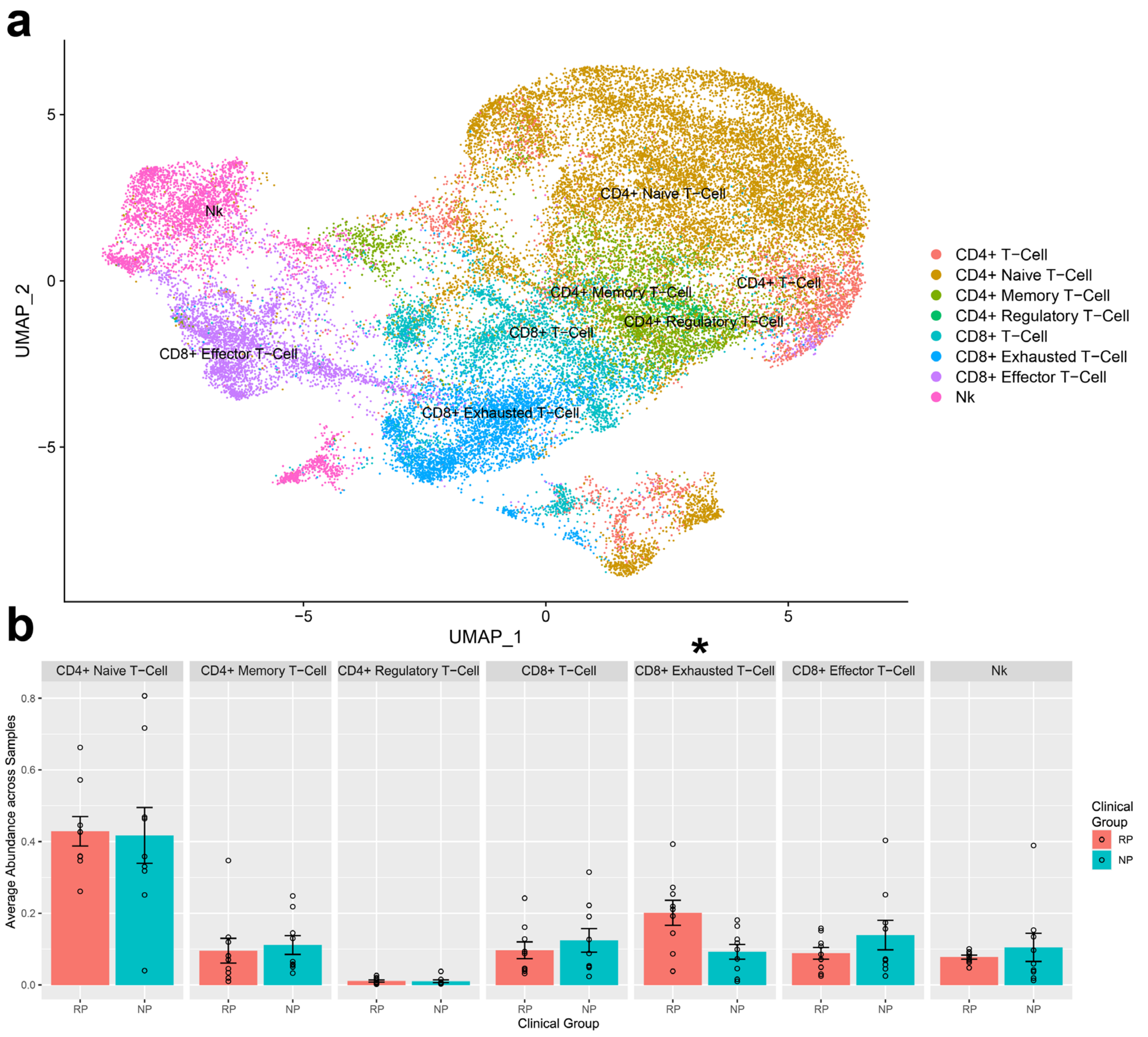

Supplementary Figure 3: Focused analysis on T cell subset after filtering out cells with >20% mitochondrial UMI. a. UMAP of T-cells following a 20% mitochondrial filter. **b.** Comparison of the average ratio of the various T-cell subtypes in RP and NP patient samples. $CD8^{+}$ Exhausted T-cells remain significantly enriched as a fraction of all T-cells (P = 0.019) with a stricter mitochondrial filter. Each bar plot depicts the mean proportion of cells within individual samples with an error bar displaying the standard error of mean. Dots represent the proportion of a given cell type in an individual sample.

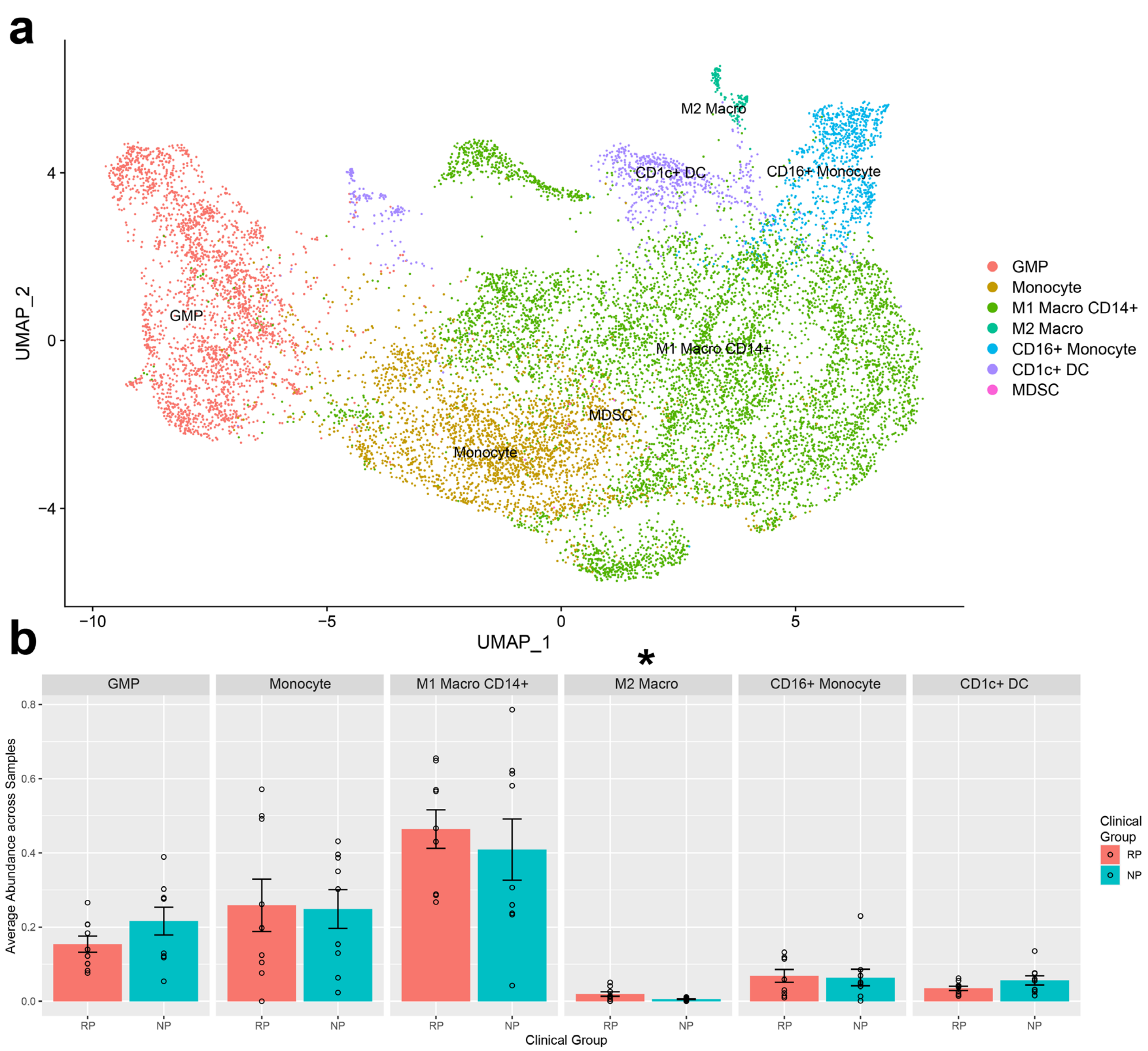

Supplementary Figure 4: Focused analysis on Monocyte and Macrophages after filtering out cells with >20% mitochondrial UMI. a. UMAP of myeloid samples following a 20% mitochondrial filter. **b.** Comparison of the average ratio of the various myeloid subtypes in RP and NP patient samples. M2 Macrophages remain significantly enriched in RPs as a fraction of all myeloid cells (P = 0.049) with a stricter mitochondrial filter. Each bar plot depicts the mean proportion of cells within individual samples with an error bar displaying the standard error of mean. Dots represent the proportion of a given cell type in an individual sample.

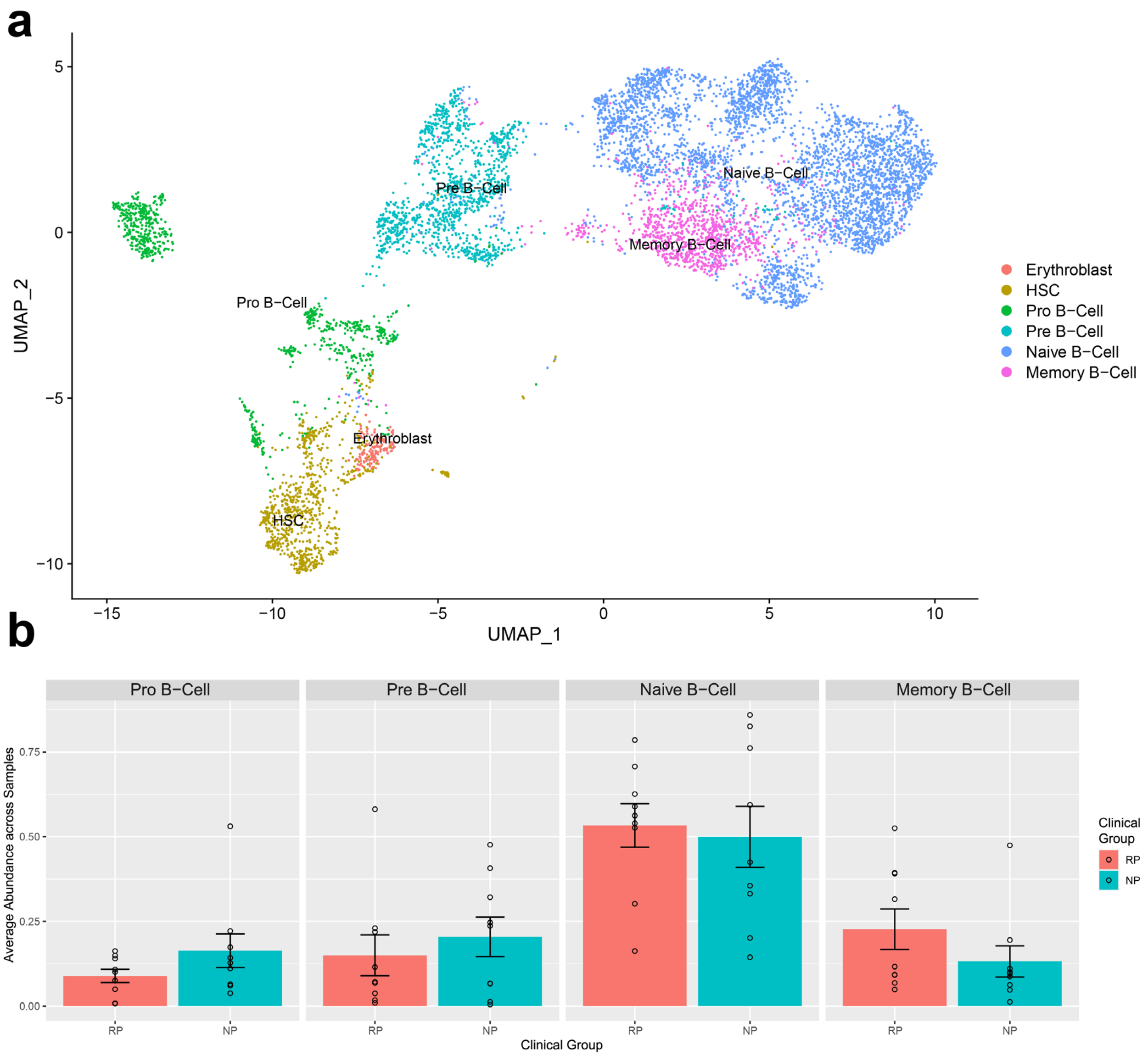

Supplementary Figure 5: Focused analysis on B- and progenitor cells after filtering out cells with >20% mitochondrial UMI. a. UMAP of B-cell and Progenitor samples following a 20% mitochondrial filter. **b.** Comparison of the average ratio of the various B-cell subtypes in RP and NP patient samples. Each bar plot depicts the mean proportion of cells within individual samples with an error bar displaying the standard error of mean. Dots represent the proportion of a given cell type in an individual sample.

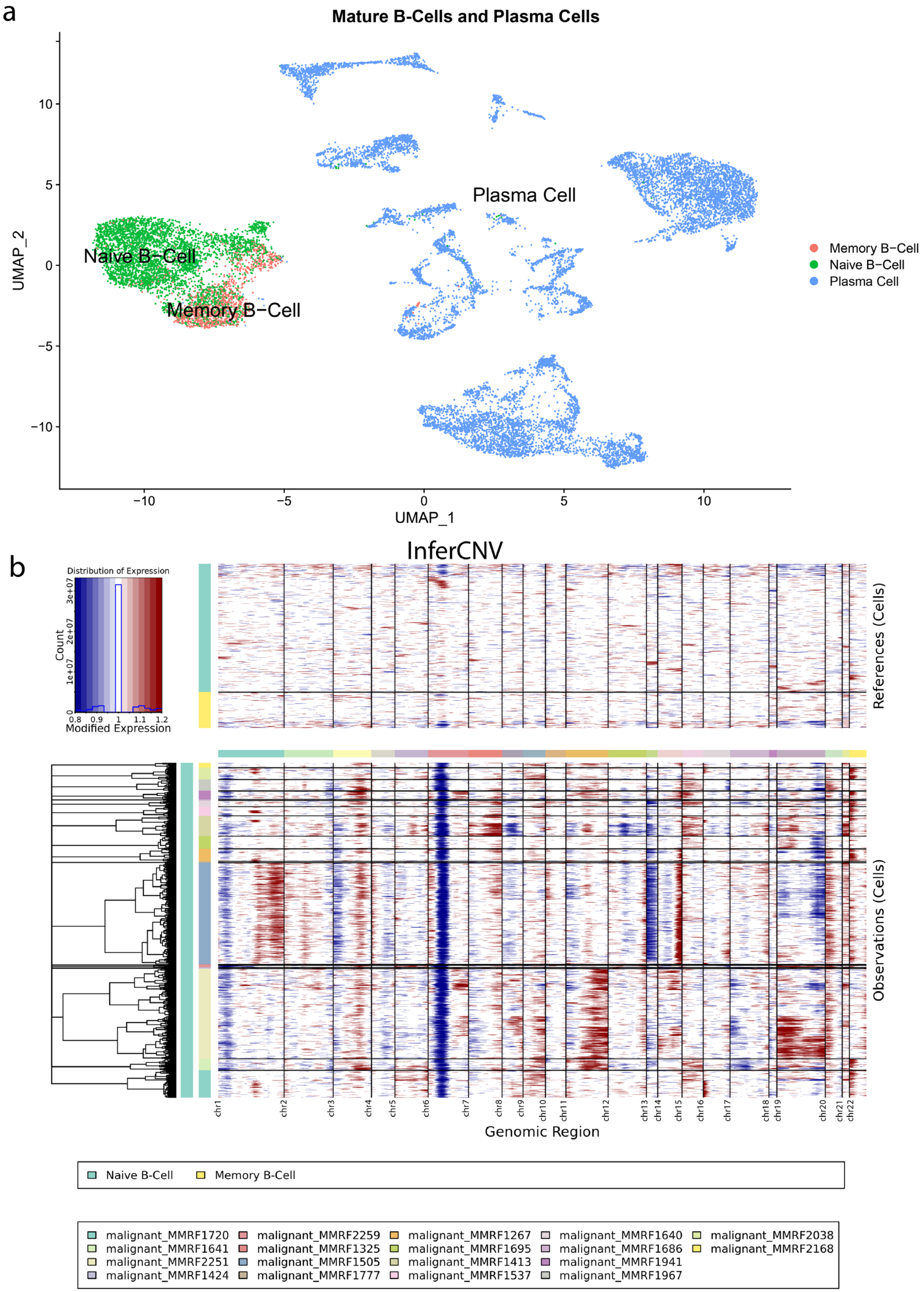

a
Mature B-Cells and Plasma Cells
Naive B−Cell
Memory B−Cell
Plasma Cell
UMAP_2
UMAP_1
10
5
0
−5
−10
−10
−5
0
5
10
Memory B−Cell
Naive B−Cell
Plasma Cell
b
InferCNV
Distribution of Expression
Count
3e+07
2e+07
1e+07
0
0.8
0.9
1
1.1
1.2
Modified Expression
References (Cells)
Observations (Cells)
chr1
chr2
chr3
chr4
chr5
chr6
chr7
chr8
chr9
chr10
chr11
chr12
chr13
chr14
chr15
chr16
chr17
chr18
chr19
chr20
chr21
chr22
Genomic Region
Naive B-Cell
Memory B-Cell
malignant_MMRF1720
malignant_MMRF1641
malignant_MMRF2251
malignant_MMRF1424
malignant_MMRF2259
malignant_MMRF1325
malignant_MMRF1505
malignant_MMRF1777
malignant_MMRF1267
malignant_MMRF1695
malignant_MMRF1413
malignant_MMRF1537
malignant_MMRF1640
malignant_MMRF1686
malignant_MMRF1941
malignant_MMRF1967
malignant_MMRF2038
malignant_MMRF2168

Supplementary Figure 6: Comparative analysis of malignant Plasma cells with normal cells in our dataset. Plasma Cells were compared with the closest normal reference population in our dataset, Naïve and Memory B-cells, to verify if technical differences could drive some of the copy number variations (CNVs) observed in the InferCNV analysis. **a.** Plasma and mature B-cells were subsetted and analyzed after normalization, variable feature selection and identifying UMAP embeddings. Plasma cells show distinct separation from the mature B-cell population and form multiple distinct subclusters. **b.** CNV analysis was performed on the plasma cells using the memory and naïve B-cell populations as a normal reference. Multiple deletions were detected across plasma cells on chromosomes 1 and 6, likely due to phenotypic differences between plasma and B-cell populations.

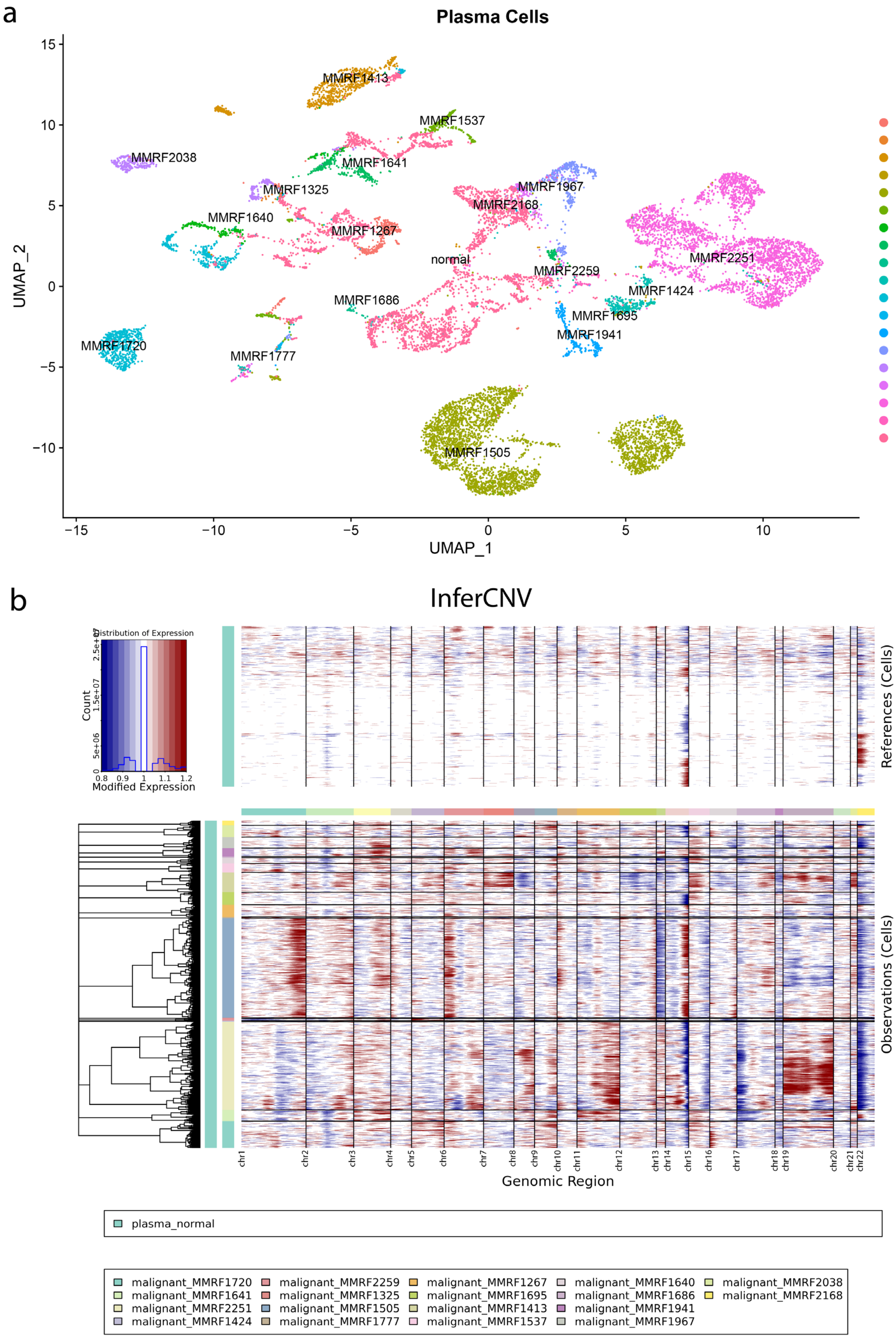
a
Plasma Cells
UMAP_1
UMAP_2
MMRF1413
MMRF1537
MMRF2038
MMRF1641
MMRF1325
MMRF1967
MMRF2168
MMRF1640
MMRF1267
normal
MMRF2251
MMRF2259
MMRF1424
MMRF1686
MMRF1695
MMRF1941
MMRF1720
MMRF1777
MMRF1505
MMRF1267
MMRF1325
MMRF1413
MMRF1424
MMRF1505
MMRF1537
MMRF1640
MMRF1641
MMRF1686
MMRF1695
MMRF1720
MMRF1777
MMRF1941
MMRF1967
MMRF2038
MMRF2168
MMRF2251
MMRF2259
normal
b
InferCNV
Distribution of Expression
Count
Modified Expression
References (Cells)
Observations (Cells)
Genomic Region
chr1
chr2
chr3
chr4
chr5
chr6
chr7
chr8
chr9
chr10
chr11
chr12
chr13
chr14
chr15
chr16
chr17
chr18
chr19
chr20
chr21
chr22
plasma_normal
malignant_MMRF1720
malignant_MMRF1641
malignant_MMRF2251
malignant_MMRF1424
malignant_MMRF2259
malignant_MMRF1325
malignant_MMRF1505
malignant_MMRF1777
malignant_MMRF1267
malignant_MMRF1695
malignant_MMRF1413
malignant_MMRF1537
malignant_MMRF1640
malignant_MMRF1686
malignant_MMRF1941
malignant_MMRF1967
malignant_MMRF2038
malignant_MMRF2168

Supplementary Figure 7: Analysis of the plasma cells from multiple myeloma samples and the human cell atlas (HCA). a. UMAP embedding of the plasma cells after merging multiple myeloma and HCA plasma cells, colored by sample source. Plasma cells are very heterogenous and show little co-embedding between datasets, or between individual patients within our multiple myeloma dataset. Plasma cells were identified via marker expression of *JCHAIN, MZB1, and SDC1* genes. A large portion of the plasma cells remaining in the CD138^{-} fractions were derived from two patients, MMRF1505 and MMRF2251. **b.** Copy number variation analysis on the plasma cells from our dataset along with the HCA plasma cells as a normal reference performed using the InferCNV algorithm. Estimated CNVs were displayed with respect to genomic region across the columns, while rows display individual cells, grouped by source. Chromosomal amplifications (red) and deletions (blue) inferred across each chromosome are represented, with MM plasma cells and normal HCA plasma cells shown in the bottom and top heatmap respectively.

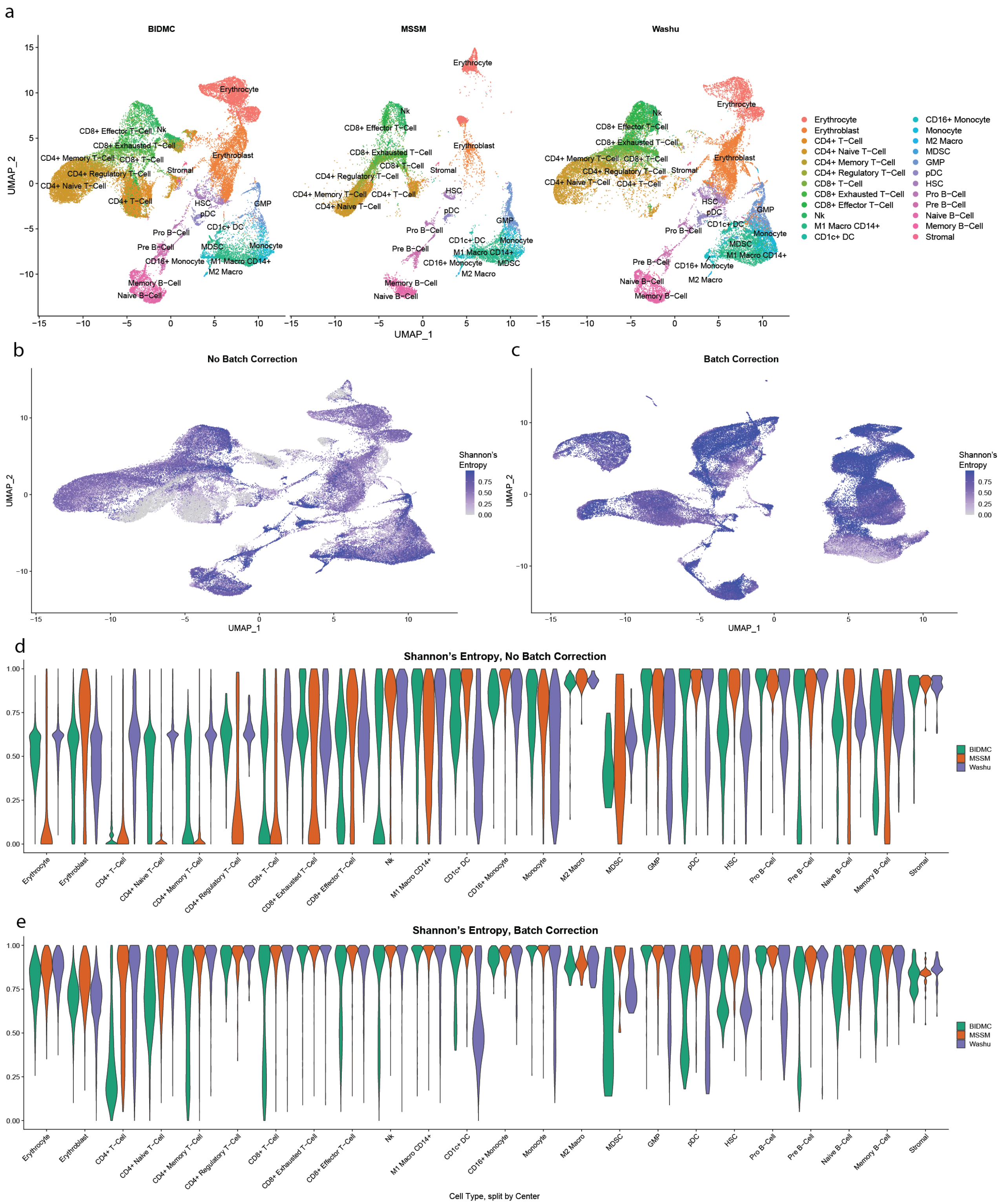

Supplementary Figure 8: Estimating the impact of different data generating centers on the single-cell profiles. **a.** UMAP embeddings of the normalized single-cell data annotated based on cell types and split by data generating centers. Major cell compartments such as T-cells, B-cells, Myeloid cells, Erythroid cells, and progenitors generally co-embed, or closely embed between all three centers, though MSSM shows some batch effect relative to BIDMC and WashU samples. **b.** The overlay of Shannon's entropy in the

UMAP plot was calculated based on the distribution of center labels among the cell's 100 nearest neighbors. The analysis was performed on normalized data without any batch effect correction. **c.** Shannon's entropy estimation after batch effect correction using integration anchors approach. **d.** Violin plots of Shannon's entropy prior to batch effect correction, grouped by cell type and split by the center. The $CD4^+$ T-cells from BIDMC and MSSM T-cells show much lower entropies, indicating a significant batch effect. **e.** Violin plots of Shannon's entropy after batch effect correction, grouped by cell type and split by the center. Most cell types show high entropy metrics, indicating better mixing of samples from different centers, though the lower quality $CD4^+$ T-cells still show low entropy, as they primarily are derived from the BIDMC samples.

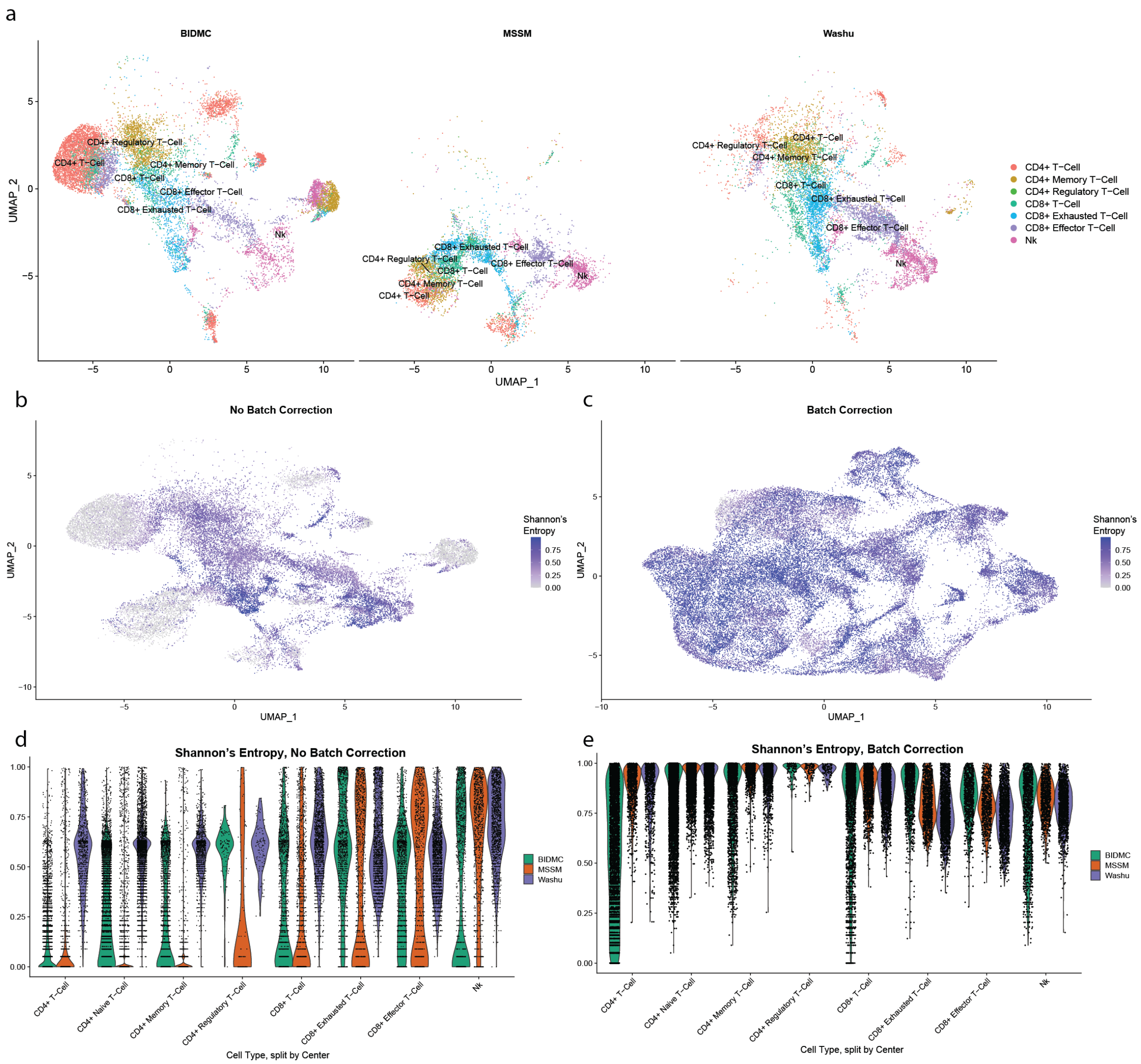

Supplementary Figure 9: Estimating the impact of different data generating centers on the single-cell profiles of T cells. a. UMAP embeddings of the normalized single-cell data of T cells without any batch correction and split based on data generating centers. $CD8^+$ T and NK cell types show somewhat similar co-embeddings among centers, though $CD4^+$ T cells show larger batch effects. Samples from BIDMC and WashU primarily differ in $CD4^+$ T-cell class, which is a lower quality subset primarily derived from BIDMC samples. **(b-c)** Shannon's entropy estimation is based on centers before (B.) and after (C.) batch correction. $CD8^+$ T cells and NK cells generally show higher entropies, while $CD4^+$ T Cells show lower entropies before batch effect correction. After batch correction, entropy is generally higher for all cell types, including the $CD4^+$ cells. **(d-e)** Violin plots of Shannon's entropy before (D) and after (E) batch correction, grouped by cell type and split by the center. Each dot represents an individual cell. $CD4^+$ T-cells show near zero entropy prior to integration, indicating large batch effects. Following integration-based batch correction, T cell subtypes show high entropy metrics, indicating better mixing of samples from

individual centers, though the lower quality of $CD4^+$ T-cells still shows low entropy, as they primarily are derived from BIDMC samples.

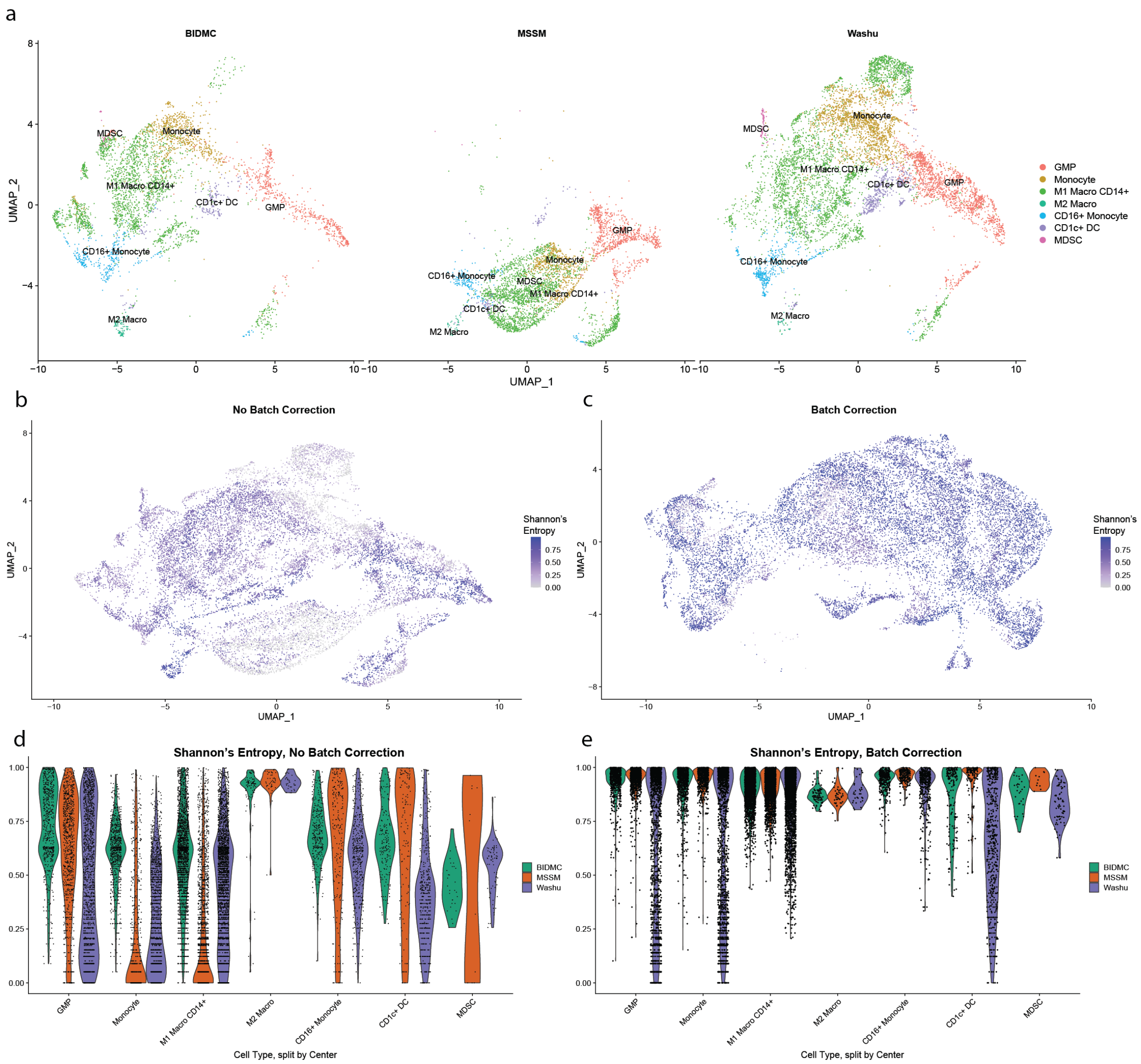

Supplementary Figure 10: Estimating the impact of different data generating centers on the single-cell profile of Monocytes and Macrophages. a. UMAP embeddings of the normalized single-cell data of Monocytes and Macrophages without any batch correction and split based on data generating centers. WashU and BIDMC show display relatively consistent co-embeddings, while MSSM M1 Macrophage and Monocyte clusters generally embed independently of other centers. **(b-c)** Shannon's entropy estimation for data from each center before (b) and after (c) batch effect correction. Shannon's entropy is generally lower for MSSM samples, and higher for WashU and BIDMC samples. Less abundant cell types, such as M2 macrophage and CD16 monocytes, co-embed and display higher entropies than more abundant cell types. Entropy increases following batch correction, with the primary exception being M2 macrophages, which showed little center-dependent batch effect prior to integration. **(d-e)** Violin plots of Shannon's entropy before (d) and after (e) batch effect correction, grouped by cell type and split by center. Each dot represents an individual cell.

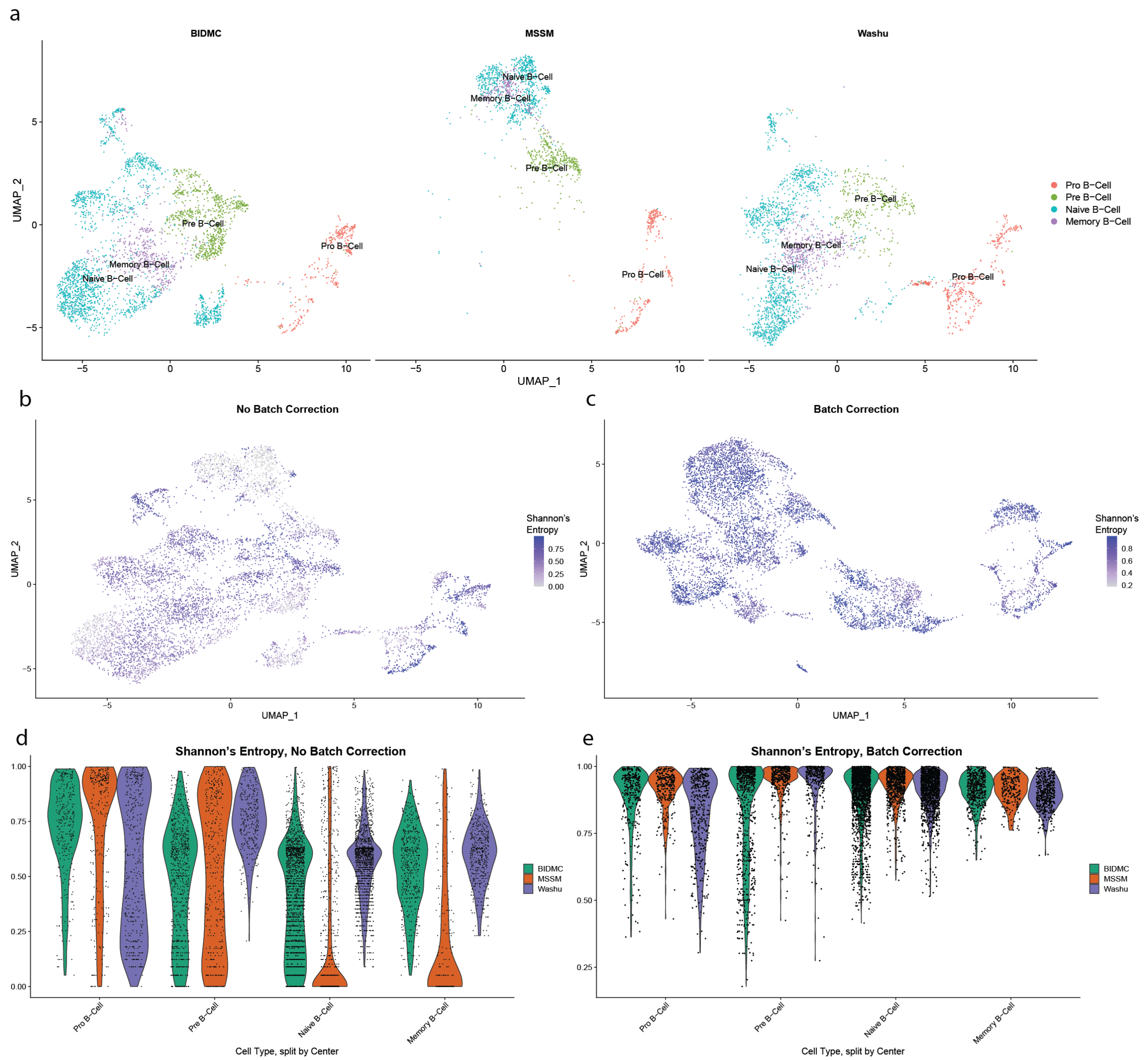

Supplementary Figure 11: Estimating the impact of different data generating centers on the single-cell profile of B cells a. UMAP embeddings of the normalized single-cell data of B cells without any batch effect correction and split based on data generating centers. Immature populations such as the Pro-B cells generally display more consistent embeddings among three centers. The mature populations show a larger batch effect between MSSM and the other two centers, with some sample-specific clusters present in comparing BIDMC to WashU samples. **(b-c)** Shannon's entropy estimation before (b) and after (c) batch correction. Shannon's entropy was higher for the less mature Pro B-cell population, while mature B-cell types tended to have lower entropies. Following integration, all B-cells display higher entropies, as expected. **(d-e)** Violin plots of Shannon's entropy before (d) and after (e) batch effect correction, grouped by cell type and split by the center. Each dot represents an individual cell.

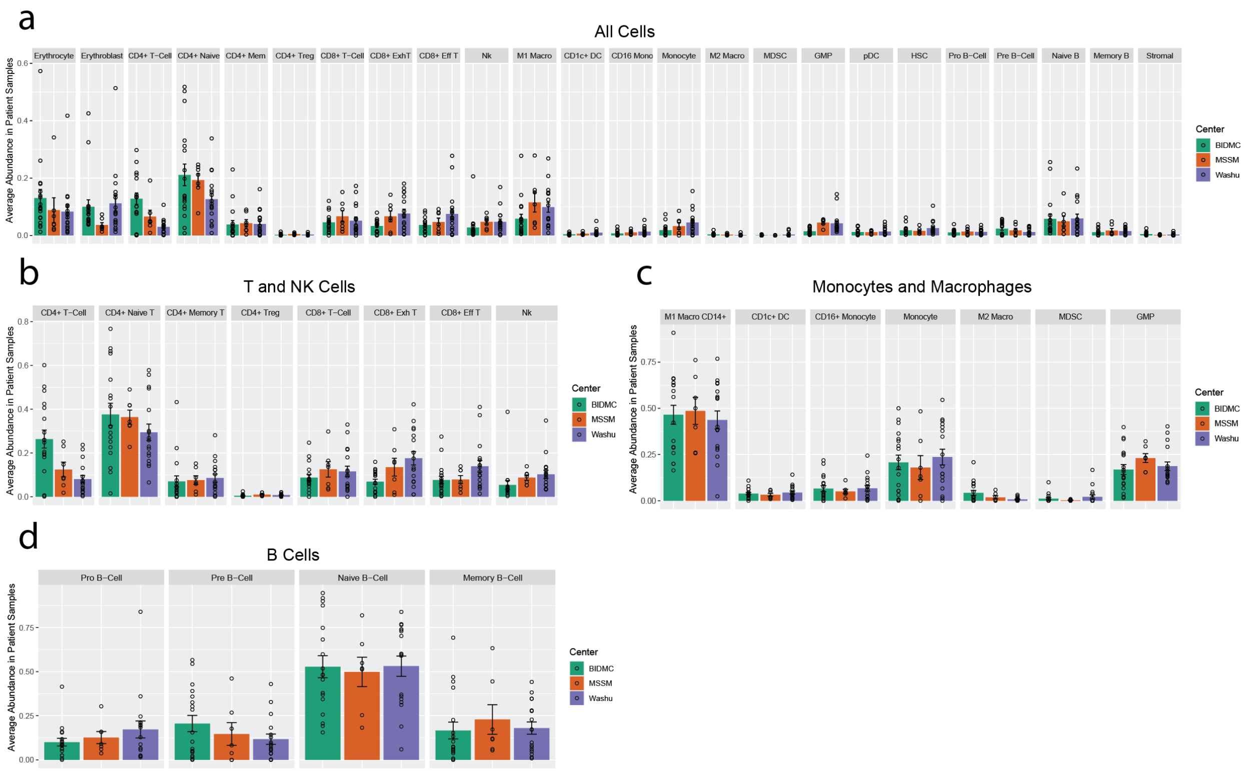

Supplementary Figure 12: Cellular abundance analysis for samples across each data generating center. Comparative analysis of cell type proportion across centers. Each bar represents the mean proportions of cells ± standard error for a given cell type for all samples processed at each center. Dots in each bar represent cell type ratios from individual patients. **a.** Cell ratios display the per-center cell type ratios for 24 major cell types identified in the study. **b.** Bar plots displaying the per-center T-cell subtype ratios. **c.** Bar plots displaying the per-center Mono/Macro subtype ratios. **d.** B-Cell subtype ratios.

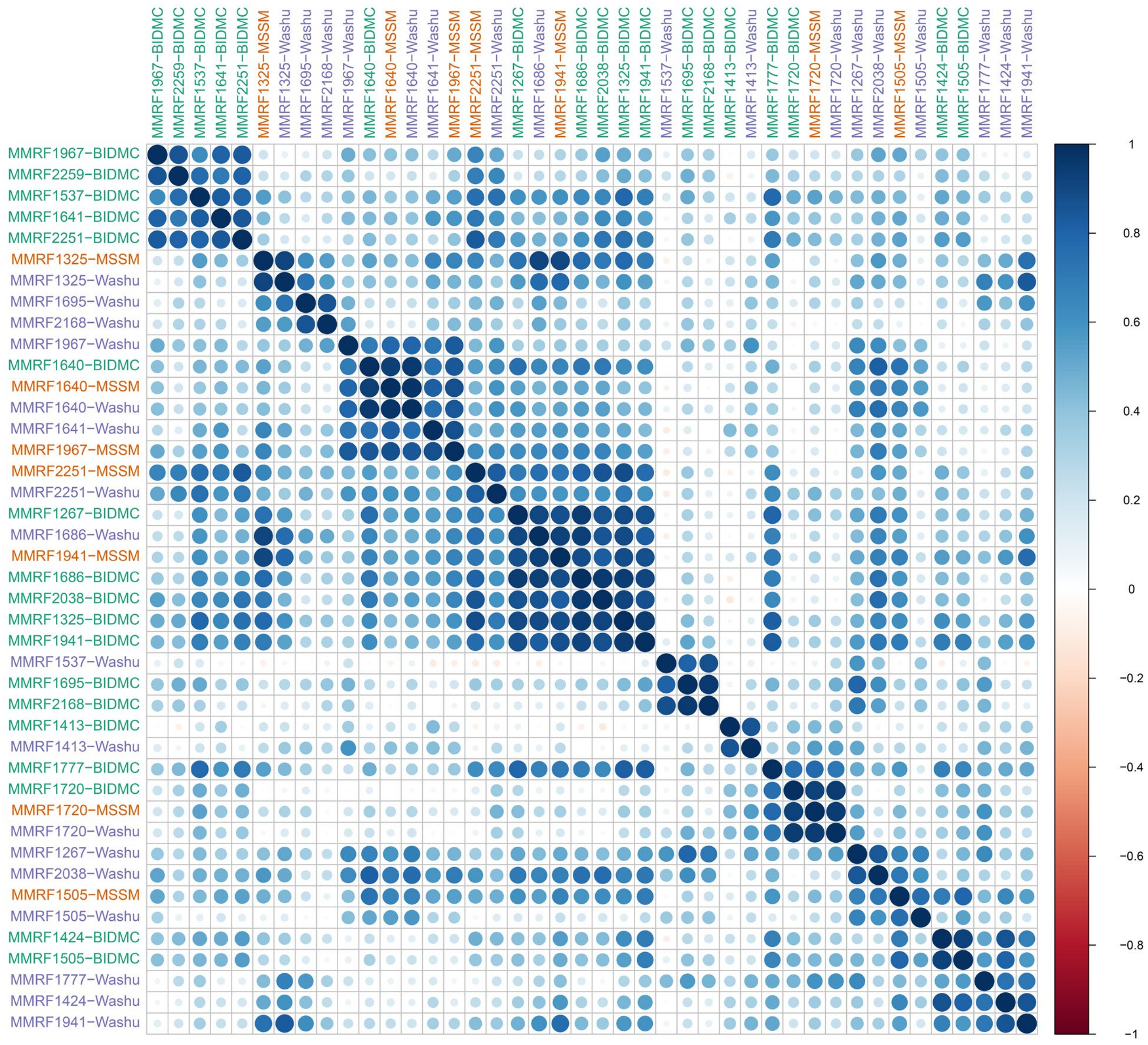

Supplementary Figure 13: Correlation among cellular enrichment from different centers. The Pearson correlation was computed between the ratios of different cell types identified at individual processing center. The correlation of cell type ratios between centers varies from sample to sample not showing any specific pattern. Some samples such as MMRF1640, MMRF1720, and MMRF1505 show high correlations across processing centers, while other samples such as MMRF2168 show weaker correlations with samples from other locations.

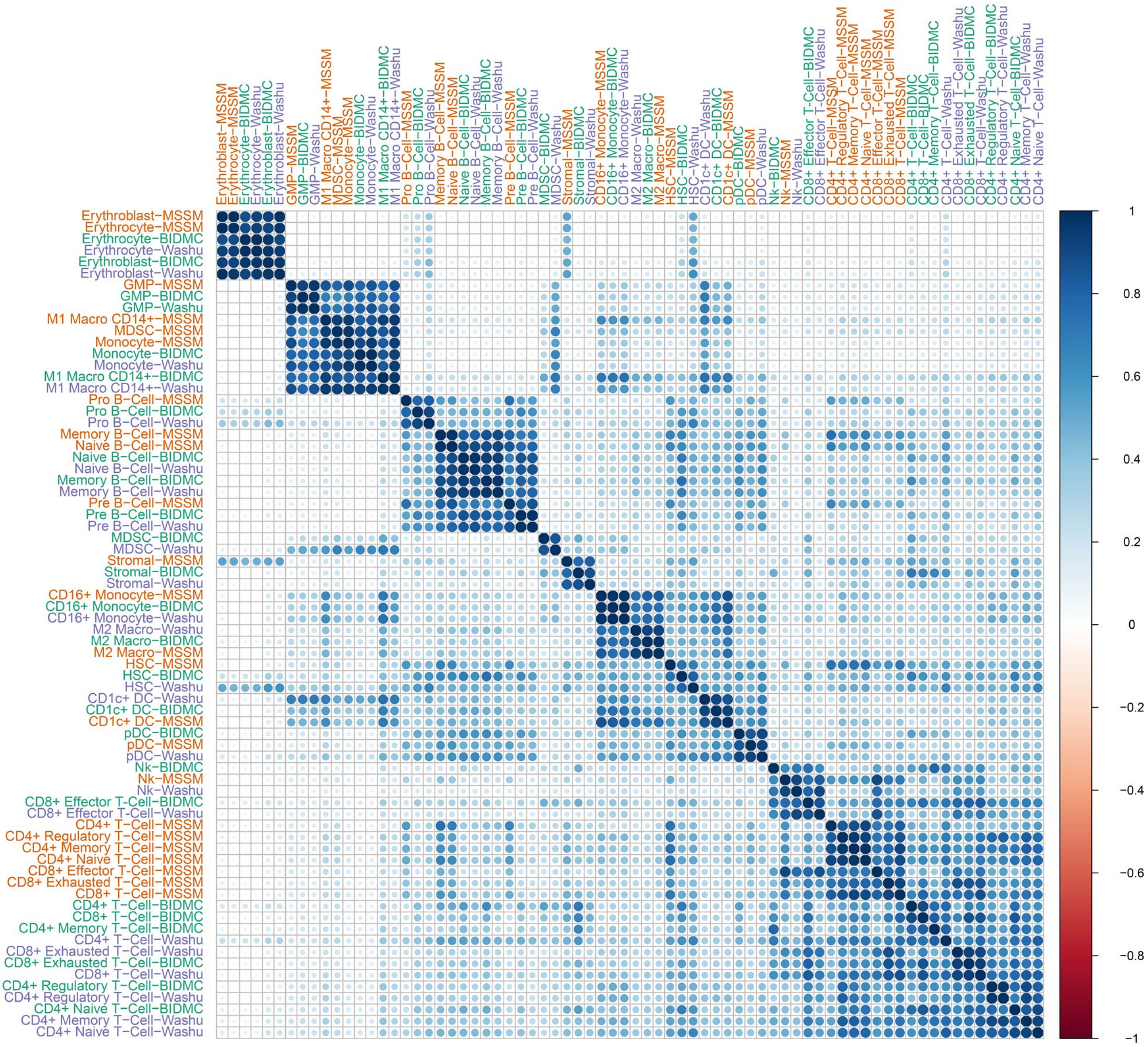

Supplementary Figure 14: Correlation of transcriptomic profiles for individual cell types across centers. Pearson Correlation of the top 2000 variable genes for all cell types across centers after normalization. Generally, the strongest correlations observed between major cell compartments (Erythroid, Myeloid, B-Cell, T-cell) irrespective of the center.

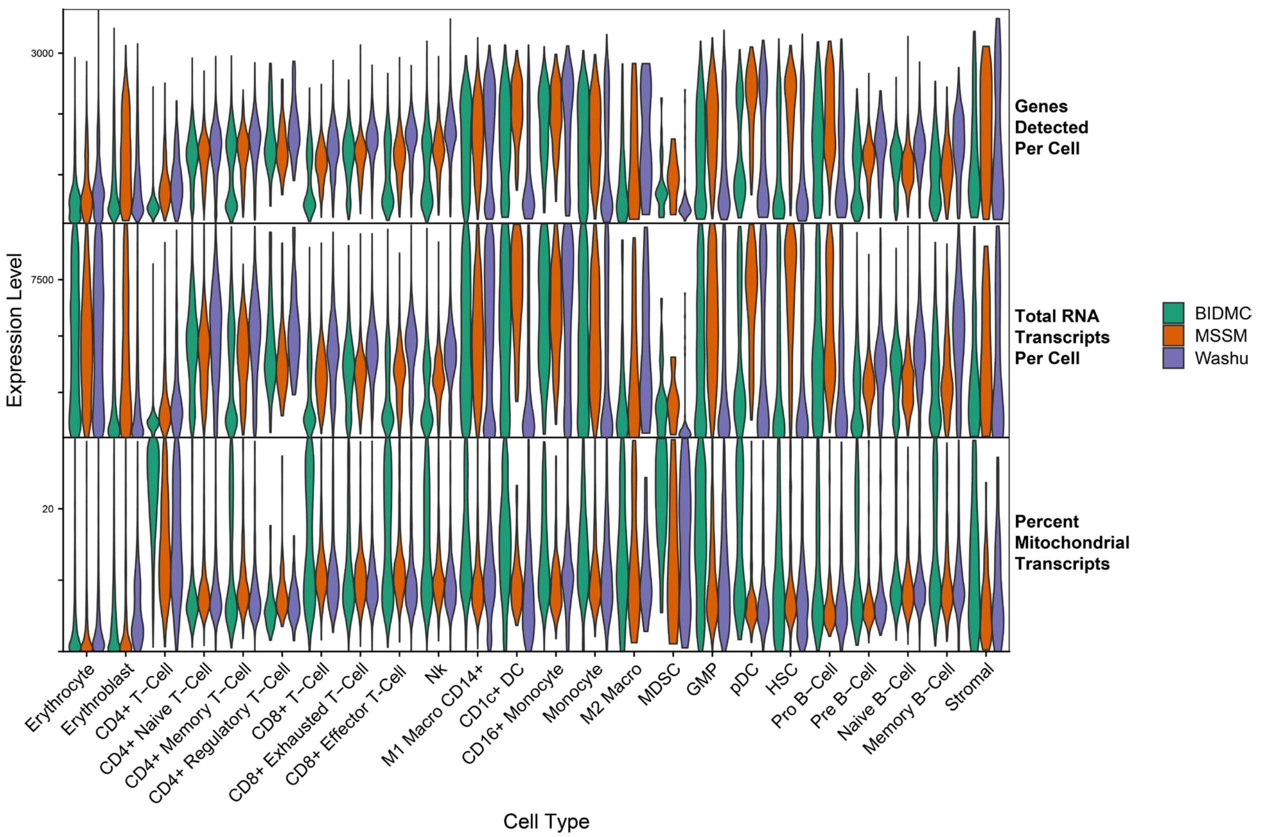

Supplementary Figure 15: Overview of single cell data standard quality metrics for each Cell Type Split based on data generating center. Violin plot displaying the quality control metrics for each cell type split by data generating centers (i.e., Washu, MSSM, BIDMC). Samples from BIDMC generally show a higher percentage of mitochondrial UMI, and a lower feature and UMI count relative to other centers. This would be consistent with lower viability, which may be due to the CITE-seq procedure performed at this center increasing processing time.

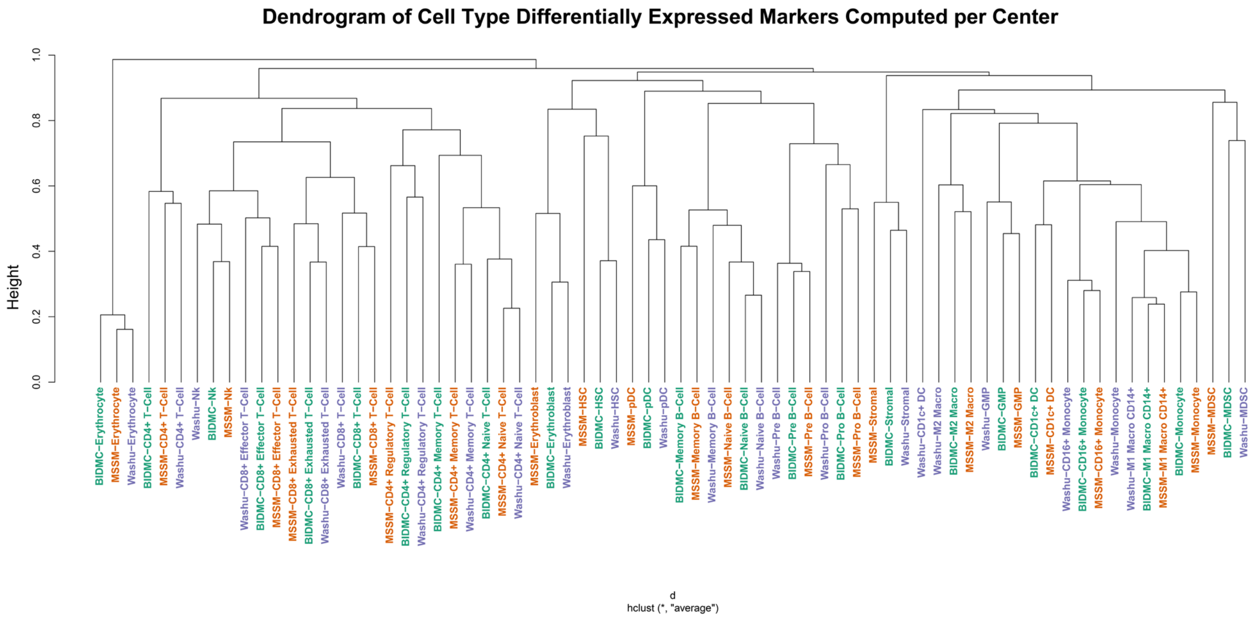

Supplementary Figure 16: Assessing the similarity of the differentially expressed cell type markers across centers. Dendrogram showing the distances between cell types from each center based on the differentially expressed genes for each cell type computed independently for each center. The differentially expressed genes were identified by comparing the target cell type with others based on log fold change >0.25 and Wilcoxon Rank Sum test P-value <0.01 as well as genes expressed in >25% of a given cell population.

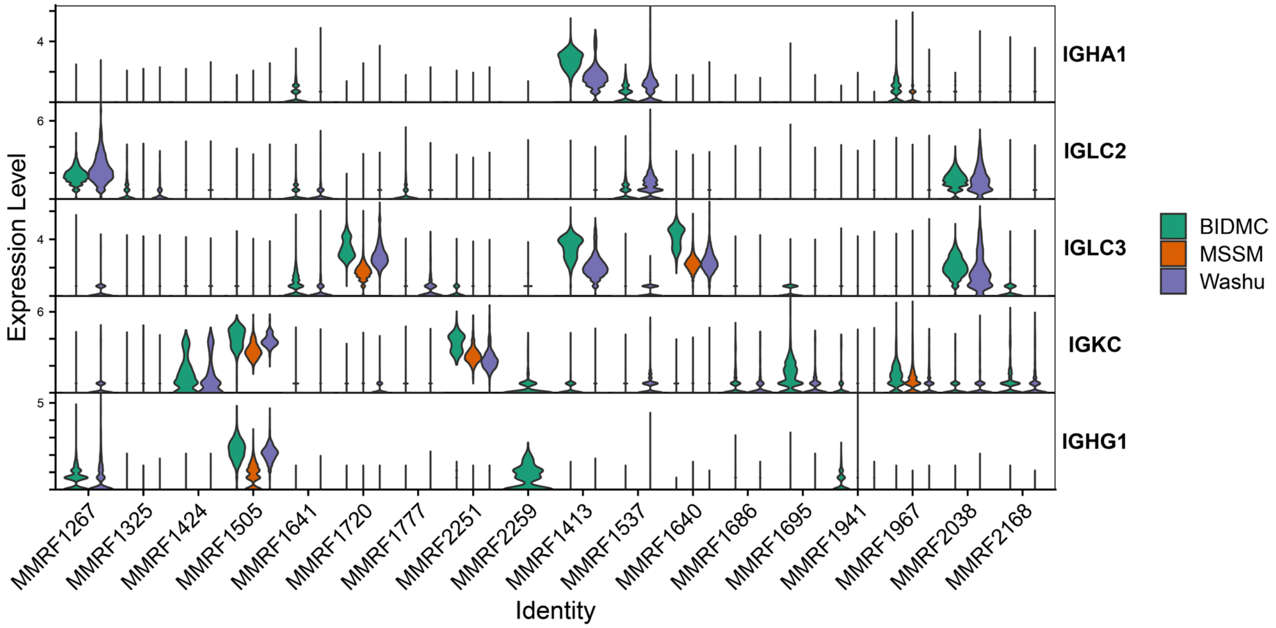

Supplementary Figure 17: Contamination of Plasma Cell estimated based on Immunoglobulin Genes Across Patients. Violin plots showing immunoglobulins gene expression in each sample separated based on the center. The presence of plasma cell contamination does not appear to be dependent on the processing center, but rather just the source of the sample.

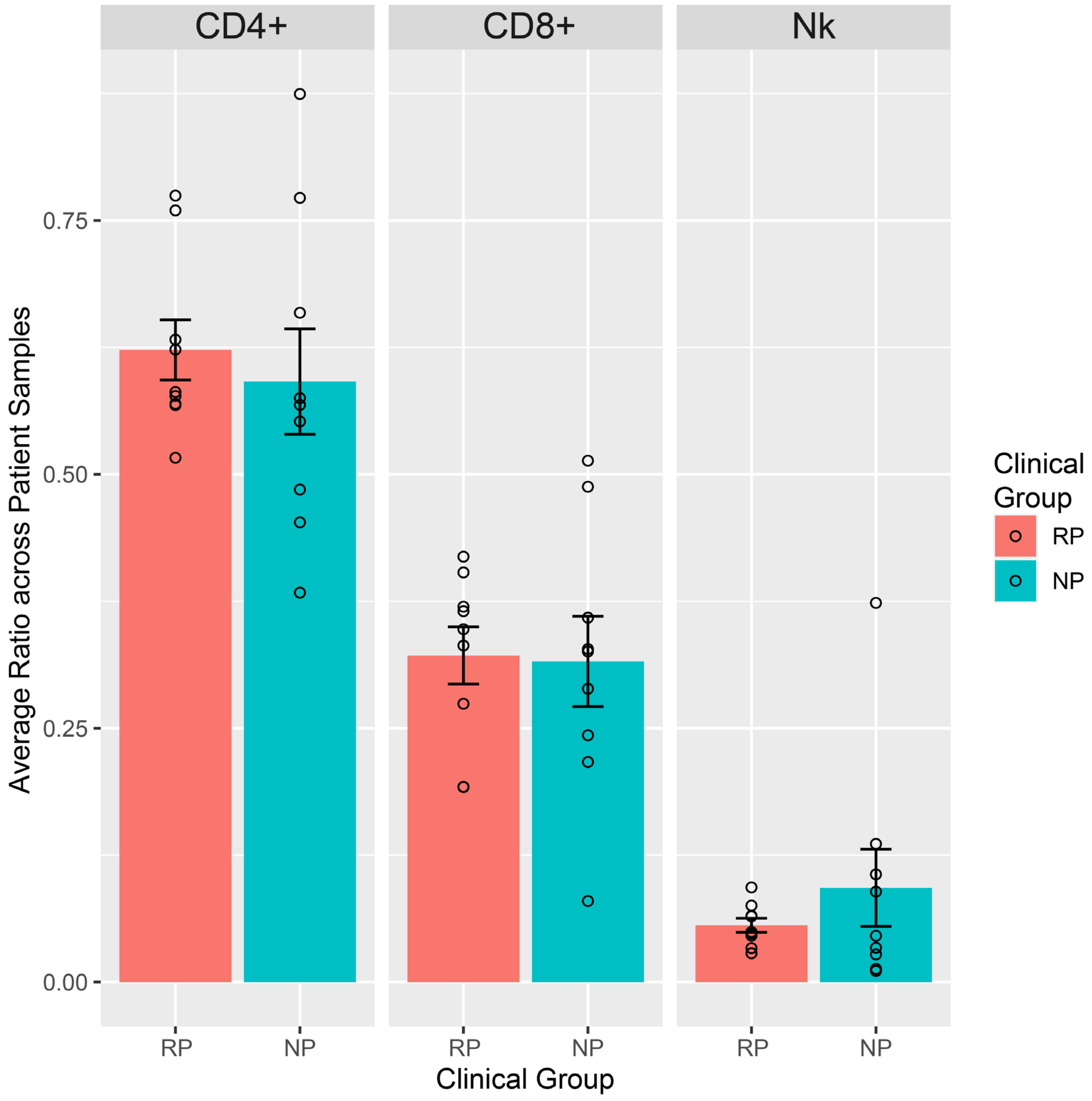

Supplementary Figure 18: Comparative analysis of major T and NK subtypes between rapid- and non-progressive groups. Comparative analysis of the CD4$^+$, CD8$^+$, and NK ratios across clinical groups. Each bar represents the mean ratio ± standard error of the mean for a given cell type across samples in a clinical group. Dots represent individual patients. There are currently no significant differences in the CD4+ T cells, CD8+ T cells, and NK cells between clinical groups.

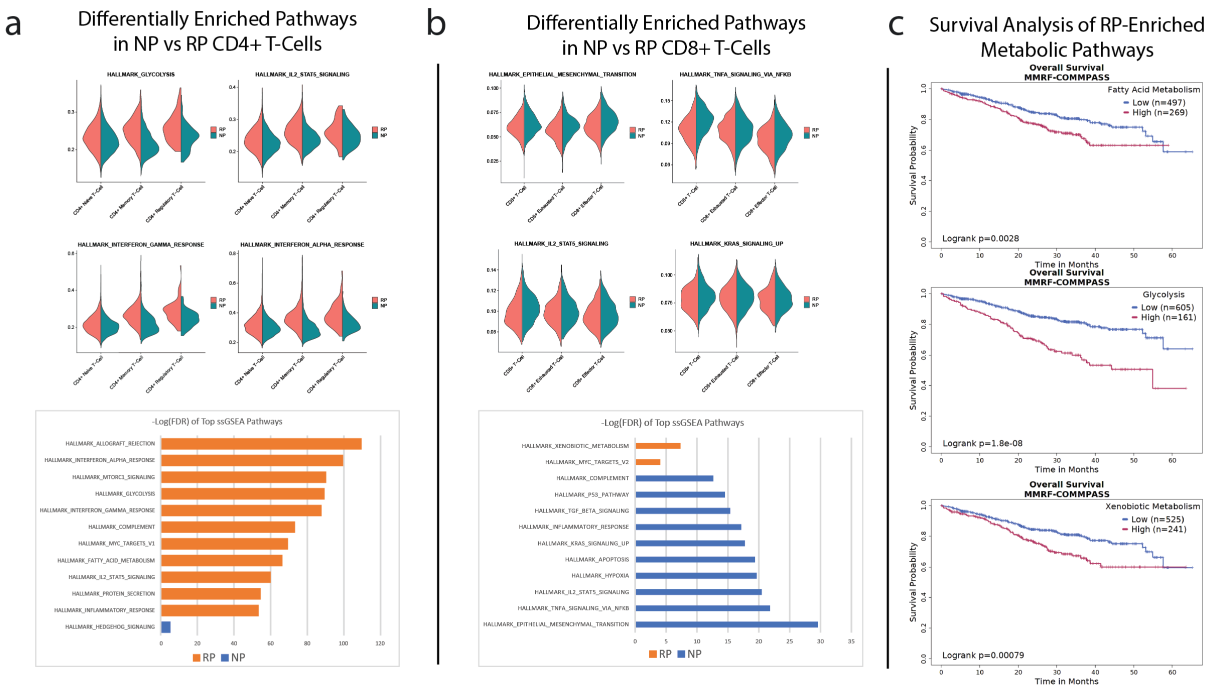

Supplementary Figure 19: Differentially Expressed Pathways between clinical groups in the T-cell subset.

a: Violin plots of top enriched pathways on the CD4^{+} subset of T-cells. Red corresponds to RP samples, while blue corresponds to NP samples. A barchart displays the negative log of the adjusted P-value for enriched pathways (adjusted P-value < 0.05), with pathways enriched in RP in orange, and NP in blue. RP samples show enrichment of various interferon signaling pathways, and some metabolic pathways, such as Glycolysis and Fatty Acid Metabolism.

b: Violin plots of enriched GSEA pathways on the CD8^{+} T-cell subsets. Red corresponds to RP samples, while blue corresponds to NP samples. A barchart displays the negative log of the adjusted P-value for subset of significantly enriched pathways (adjusted P-value < 0.05), with pathways enriched in RP in orange, and NP in blue. NP samples show enrichment of TNF signaling, while RP samples show enrichment of Xenobiotic Metabolism.

c: Survival analysis performed using Survival Genie platform against the MMRF CoMMpass dataset on the genes associated with the metabolic pathways enriched in RP samples. Enrichment of genes associated with Fatty Acid Metabolism, Glycolysis, and Xenobiotic Metabolism are all significantly associated with poorer survival ($P = 0.0028$, $P = 1.8*10^{-8}$, $P = 0.00079$).

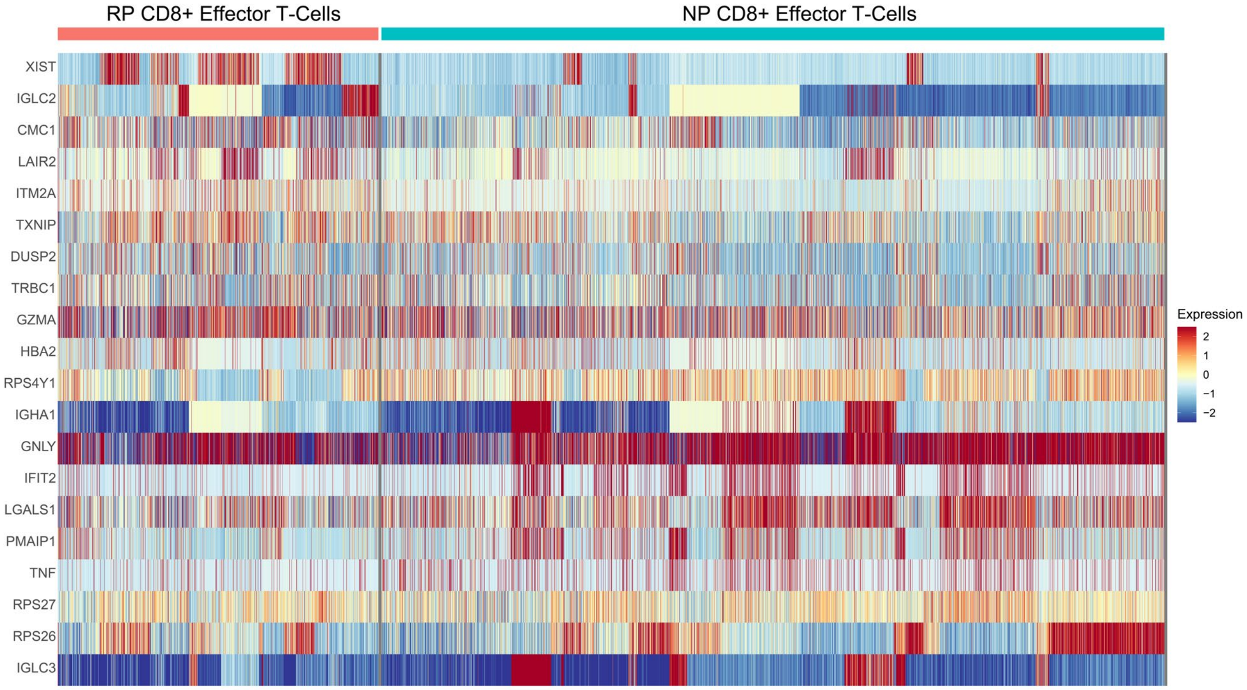

Supplementary Figure 20: Differentially Expressed Genes between Clinical Groups in CD8+ Effector T-Cells. A heatmap displaying the top differentially expressed genes between NP and RP groups within the CD8+ Effector T-Cell population. NP Effector cells show visibly more consistent expression of *TNF*, potentially indicating better cytokine and more typical effector function. Top markers show significant enrichment ($P < 0.001$) of effector genes, such as *GZMB*, *GNLY*, and *TNF* genes in the NP population (**Supplementary Table 2**).

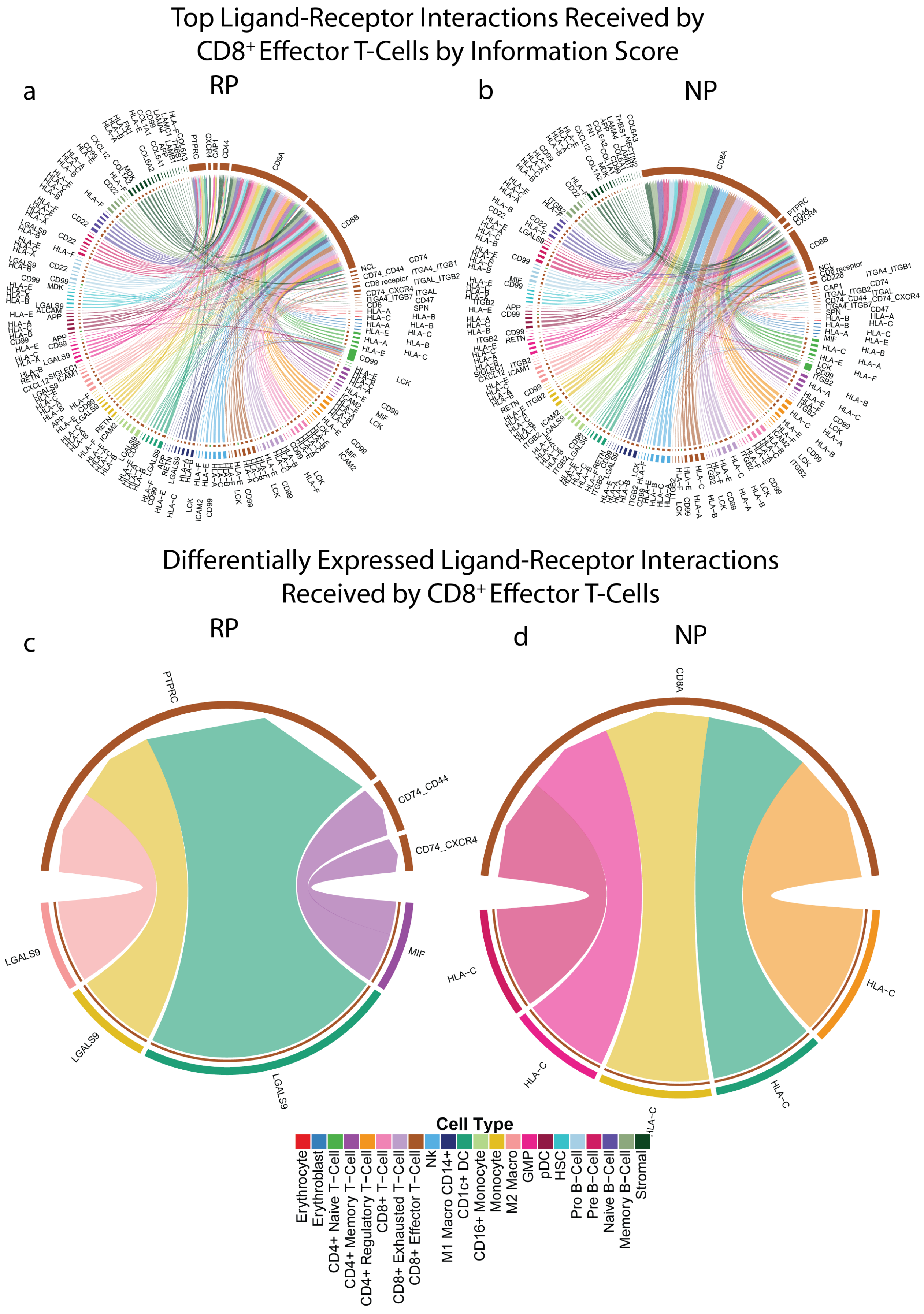
Top Ligand-Receptor Interactions Received by
CD8+ Effector T-Cells by Information Score
a
RP
b
NP
Differentially Expressed Ligand-Receptor Interactions
Received by CD8+ Effector T-Cells
c
RP
PTPRC
CD74_CD44
CD74_CXCR4
MIF
LGALS9
LGALS9
LGALS9
d
NP
CD8A
HLA-C
HLA-C
HLA-C
HLA-C
Cell Type
Erythrocyte
Erythroblast
CD4+ Naive T-Cell
CD4+ Memory T-Cell
CD4+ Regulatory T-Cell
CD8+ T-Cell
CD8+ Exhausted T-Cell
CD8+ Effector T-Cell
Nk
M1 Macro CD14+
CD1c+ DC
CD16+ Monocyte
Monocyte
M2 Macro
GMP
pDC
HSC
Pro B-Cell
Pre B-Cell
Naive B-Cell
Memory B-Cell
Stromal

Supplementary Figure 21: Ligand-Receptor Interactions Received by CD8+ Effector T-Cells in Rapid and Non-progressing Samples. Circos plots comparing ligand-receptor interactions for CD8+ Effector T-Cells cells between the RP and NP population. The predominant signaling interaction appears to be from MHC-I – CD8 signaling. **a:** The top ligand-receptor interactions received by RP CD8+ Effector T-Cells, based on information flow. **b:** The top ligand-receptor interactions received by NP CD8+ Effector T-Cells, based on information flow. **c:** The differentially expressed ligand-receptor interactions received by RP CD8+ Effector T-cells. The only enriched signaling received by RP effector cells appears to be CD45-LGALS9 interactions. **d:** The differentially expressed ligand-receptor interactions received by NP CD8+ Effector T-cells cells. MHC-I – CD8 interactions are enriched in NP effector cells from multiple sender cell populations.

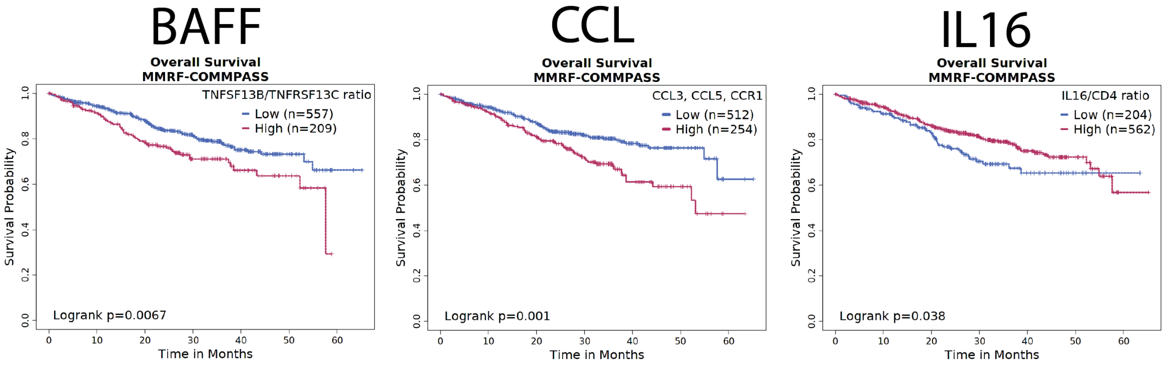

Supplementary Figure 22: Survival Analysis of Select Ligands and Receptors Identified cellular communication analysis. Survival analysis of BAFF, IL16, and CCL signaling in the MMRF CoMMpass dataset provided using the Survival Genie platform. The overexpression of BAFF ligand *TNFSF13B* relative to the typical B-Cell receptor, *TNFRSF13C*, is associated with poorer outcomes (P = 0.0067). General enrichment of ligands and receptors in CCL signaling (*CCL3*, *CCL5*, *CCR1*) is associated with poorer survival (P = 0.01). Overexpression of IL16 relative to CD4 within the Myeloma cell compartment is associated with better survival (P = 0.038).

Patient	*Clinical Group*	*Age (At Diagnosis)*	*Gender*	*Race*	*ISS*	*BIDMC*	*MSSM*	*WashU*	*IMWG Risk Class*	*ECOG Assessment*	*Gene Fusions*
MMRF1267	**RP**	**39**	**Male**	**White**	**2**	**Y**		**Y**	**2**	**1**	**Not Available**
MMRF1325	**RP**	**58**	**Female**	**Other**	**3**	**Y**	**Y**	**Y**	**1**	**1**	**MALAT1-IGL@-ext, B2M--IGL@-ext, FOSB--KLF2, FOSB--UBC, TSMC3-AS1--AC023034.1**
MMRF1424	**RP**	**40**	**Female**	**White**	**3**	**Y**		**Y**	**2**	**1**	**ERLEC1--ACYP2, PRKCB--SCNN1B, AC109349.1--LINC02472, RHOXF1-AS1--NKAPP1, IGH-@-ext--AC244205.1**
MMRF1505	**RP**	**62**	**Male**	**White**	**3**	**Y**	**Y**	**Y**	**2**	**1**	**Not Available**
MMRF1641	**RP**	**70**	**Male**	**White**	**3**	**Y**		**Y**	**2**	**3**	**IGH@-ext--AC245060.7, IGH@--AC245060.7, RYR1--SOHLH2, AC068413.1--CCDC26, PPP3CA--LINC01524**
MMRF1720	**RP**	**83**	**Male**	**White**	**3**	**Y**	**Y**	**Y**	**1**	**2**	**MTND2P28--IGL@-ext, AL162253.2--PLGRKT, AC005394.1--AC005381.1, AC093639.2--AC020584.1,**

											IGL-@-ext--PFKL
MMRF1777	**RP**	**67**	**Female**	**White**	**1**	**Y**		**Y**	**0**	**2**	**IGH-@-ext--TXNDC5, MTND2P28--IGL@-ext, AL138963.4--IGL@-ext, C12orf73--IGL@-ext, IGH-@-ext--SPON2**
MMRF2251	**RP**	**47**	**Female**	**White**	**3**	**Y**		**Y**	**2**	**1**	**MRPS16--CFAP70, ATP11C--MCF2**
MMRF2259	**RP**	**50**	**Female**	**Black**	**3**	**Y**			**Not Available**	**1**	**Not Available**
MMRF1413	**NP**	**71**	**Male**	**White**	**3**	**Y**		**Y**	**1**	**1**	**IGH-@--EEF1A1, CREB3L2--DGKI, AC009570.2--IGL@-ext, B2M--KLF2, STK25--IGH@-ext**
MMRF1537	**NP**	**62**	**Male**	**White**	**3**	**Y**		**Y**	**1**	**1**	**JCHAIN--IGH@-ext, IGL-@-ext--PRAMENP, PLCXD1--IGH@-ext, IGL-@-ext--DGKG**
MMRF1640	**NP**	**71**	**Male**	**White**	**3**	**Y**	**Y**	**Y**	**1**	**1**	**OAZ1--KLF2, ZFP36--KLF2**
MMRF1686	**NP**	**38**	**Female**	**White**	**1**	**Y**		**Y**	**0**	**1**	**CD40--IGL@**
MMRF1695	**NP**	**61**	**Male**	**White**	**3**	**Y**		**Y**	**1**	**Not Available**	**IGH-@-ext--EEF1A1P5, IGH-@-ext--TXNDC5,**

											IGH@-ext--AC244205.1, FOSB--KLF2, IGL-@-ext--PRAMENP
MMRF1941	**NP**	**59**	**Male**	**White**	**2**	**Y**	**Y**	**Y**	**Not Available**	**0**	**IGH@-ext--NSD2, FOSB--KLF2, AC087280.2--DCHS1, AL391840.3--SH3BGRL2, GPIHBP1--IGL@-ext**
MMRF1967	**NP**	**74**	**Male**	**White**	**2**	**Y**	**Y**	**Y**	**0**	**0**	**PAX5--IGH@-ext, DDX42--TANC2, AC106741.1--AC106729.1, AL391840.3--SH3BGRL2,**
MMRF2038	**NP**	**37**	**Female**	**White**	**3**	**Y**		**Y**	**1**	**3**	**NFKB2--TRIM8, AC093639.2--AC020584.1, C12orf73--IGL@-ext, FOSB--KLF2, OAZ1--KLF2**
MMRF2168	**NP**	**50**	**Male**	**Black**	**1**	**Y**		**Y**	**0**	**0**	**AC244205.1--IGH@-ext, IGL-@-ext--PRAMENP, SNX22--IGH@-ext, FOS--IGH@-ext, IGH@--SIK1**

Supplementary Table 1: Demographic, clinical, and genetic information about the 18 patients included in the study, along with information about sample processing centers.

GENE	P_VAL	AVG_LOG2FC	PCT.1	PCT.2	P_VAL_ADJ	CLUSTER	GENE
**XIST**	4.73E-191	0.957753	0.41	0.052	8.39E-187	RP	XIST
**IGLC2**	9.51E-64	0.43629	0.245	0.063	1.69E-59	RP	IGLC2
**CMC1**	4.81E-38	0.479971	0.448	0.261	8.54E-34	RP	CMC1
**LAIR2**	1.10E-35	0.271827	0.208	0.076	1.95E-31	RP	LAIR2
**ITM2A**	2.75E-30	0.260403	0.325	0.172	4.88E-26	RP	ITM2A
**TXNIP**	7.37E-14	0.293082	0.953	0.957	1.31E-09	RP	TXNIP
**DUSP2**	8.95E-14	0.225641	0.493	0.376	1.59E-09	RP	DUSP2
**GZMK**	1.15E-08	0.20959	0.227	0.155	0.000204	RP	GZMK
**TRBC1**	1.55E-06	0.241907	0.445	0.387	0.027435	RP	TRBC1
**GZMA**	1.11E-05	0.209655	0.76	0.759	0.196886	RP	GZMA
**HBA2**	0.001141	0.383479	0.244	0.199	1	RP	HBA2
**RPS4Y1**	3.17E-116	0.758573	0.688	0.303	5.63E-112	NP	RPS4Y1
**IGHA1**	2.23E-33	0.925809	0.226	0.077	3.95E-29	NP	IGHA1
**GNLY**	4.77E-32	0.553786	0.784	0.655	8.47E-28	NP	GNLY
**DDX3Y**	5.08E-32	0.286273	0.368	0.182	9.01E-28	NP	DDX3Y
**IFIT2**	2.45E-29	0.855113	0.233	0.095	4.35E-25	NP	IFIT2
**LGALS1**	2.99E-29	0.519459	0.566	0.414	5.30E-25	NP	LGALS1
**PMAIP1**	2.64E-23	0.66558	0.356	0.228	4.69E-19	NP	PMAIP1
**CD8A**	6.27E-23	0.31959	0.524	0.365	1.11E-18	NP	CD8A
**RPS27A**	4.07E-20	0.252505	1	0.998	7.22E-16	NP	RPS27A
**RPL26**	3.55E-19	0.26874	0.996	0.991	6.30E-15	NP	RPL26
**RPL39**	5.09E-19	0.27942	0.999	0.996	9.03E-15	NP	RPL39
**TNF**	7.57E-18	0.355121	0.202	0.098	1.34E-13	NP	TNF
**RPLP1**	2.00E-16	0.243544	1	0.998	3.55E-12	NP	RPLP1
**RPL34**	4.12E-16	0.248138	1	0.997	7.31E-12	NP	RPL34
**RPS27**	4.15E-16	0.331514	1	0.998	7.36E-12	NP	RPS27
**NFKBIA**	1.23E-15	0.275355	0.747	0.666	2.18E-11	NP	NFKBIA
**RPS15A**	1.62E-15	0.22181	0.999	0.996	2.87E-11	NP	RPS15A
**RPL23A**	5.28E-15	0.244312	0.998	0.995	9.37E-11	NP	RPL23A
**RPL14**	9.05E-15	0.208288	0.997	0.993	1.61E-10	NP	RPL14
**RPS14**	9.28E-15	0.209837	1	0.998	1.65E-10	NP	RPS14
**RPS12**	1.05E-14	0.217714	0.998	0.996	1.86E-10	NP	RPS12
**ITGB1**	1.25E-14	0.237638	0.598	0.483	2.22E-10	NP	ITGB1
**RPL37A**	2.13E-14	0.26619	0.997	0.992	3.78E-10	NP	RPL37A
**CD52**	2.99E-14	0.309449	0.848	0.788	5.30E-10	NP	CD52
**PPP1R15A**	5.78E-14	0.26897	0.544	0.428	1.03E-09	NP	PPP1R15A
**RPS3**	6.88E-14	0.202415	1	0.996	1.22E-09	NP	RPS3
**JUND**	8.25E-14	0.231484	0.934	0.923	1.46E-09	NP	JUND
**RPL41**	2.40E-13	0.224582	1	0.998	4.25E-09	NP	RPL41
**ANXA1**	2.93E-13	0.264589	0.753	0.66	5.20E-09	NP	ANXA1
**CRIP1**	4.53E-13	0.259311	0.409	0.308	8.03E-09	NP	CRIP1
**TNFAIP3**	5.29E-13	0.308608	0.437	0.337	9.39E-09	NP	TNFAIP3

**S100A10**	5.65E-13	0.26638	0.738	0.661	1.00E-08	NP	S100A10
**RPL21**	8.24E-13	0.224668	0.998	0.995	1.46E-08	NP	RPL21
**RPS21**	1.13E-12	0.207394	0.988	0.973	2.01E-08	NP	RPS21
**FGFBP2**	2.02E-12	0.265006	0.638	0.539	3.59E-08	NP	FGFBP2
**RPS18**	2.76E-12	0.202983	0.999	0.996	4.89E-08	NP	RPS18
**CYTOR**	9.74E-12	0.223619	0.391	0.292	1.73E-07	NP	CYTOR
**S100A4**	2.13E-11	0.241591	0.887	0.849	3.78E-07	NP	S100A4
**RPL37**	2.89E-11	0.228638	0.998	0.995	5.13E-07	NP	RPL37
**FOS**	1.10E-10	0.309332	0.615	0.529	1.95E-06	NP	FOS
**RPS26**	1.53E-10	0.541942	0.85	0.809	2.71E-06	NP	RPS26
**TRBC2**	1.72E-10	0.22511	0.64	0.57	3.05E-06	NP	TRBC2
**GZMB**	2.01E-10	0.221009	0.593	0.501	3.57E-06	NP	GZMB
**KLF6**	2.94E-10	0.265233	0.871	0.842	5.22E-06	NP	KLF6
**RPS28**	3.04E-10	0.201065	0.998	0.991	5.39E-06	NP	RPS28
**RPS29**	2.96E-08	0.215475	0.992	0.984	0.000525	NP	RPS29
**IGLC3**	4.79E-07	1.406228	0.222	0.175	0.008495	NP	IGLC3
**DUSP1**	8.37E-07	0.207332	0.653	0.582	0.014847	NP	DUSP1

Supplementary Table 2: Top Differentially Expressed Genes Between Rapid and Non-Progressors in the CD8+ Effector T-cell Population.

REAGENT or RESOURCE	SOURCE	IDENTIFIER
Antibodies		
TotalSeq™-B 0005 anti-human CD80	Biolegend totalseq B	305241
TotalSeq™-B 0006 anti-human CD86	Biolegend totalseq B	305445
TotalSeq™-B 0007 anti-human CD274 (B7-H1,PD-L1)	Biolegend totalseq B	329749
TotalSeq™-B 0034 anti-human CD3	Biolegend totalseq B	300477
TotalSeq™-B 0050 anti-human CD19	Biolegend totalseq B	302263
TotalSeq™-B 0063 anti-human CD45RA	Biolegend totalseq B	304161
TotalSeq™-B 0072 anti-human CD4	Biolegend totalseq B	300565
TotalSeq™-B 0080 anti-human CD8a	Biolegend totalseq B	301069
TotalSeq™-B 0081 anti-human CD14	Biolegend totalseq B	301857
TotalSeq™-B 0083 anti-human CD16	Biolegend totalseq B	302063
TotalSeq™-B 0084 anti-human CD56 (NCAM) Recombinant	Biolegend totalseq B	392423
TotalSeq™-B 0085 anti-human CD25	Biolegend totalseq B	302647
TotalSeq™-B 0087 anti-human CD45RO	Biolegend totalseq B	304257
TotalSeq™-B 0088 anti-human CD279 (PD-1)	Biolegend totalseq B	329961
TotalSeq™-B 0089 anti-human TIGIT (VSTM3)	Biolegend totalseq B	372727
TotalSeq™-B 0100 anti-human CD20	Biolegend totalseq B	302361
TotalSeq™-B 0101 anti-human CD335 (NKp46)	Biolegend totalseq B	331939
TotalSeq™-B 0146 anti-human CD69	Biolegend totalseq B	310949
TotalSeq™-B 0147 anti-human CD62L	Biolegend totalseq B	304849
TotalSeq™-B 0148 anti-human CD197 (CCR7)	Biolegend totalseq B	353249
TotalSeq™-B 0154 anti-human CD27	Biolegend totalseq B	302851
TotalSeq™-B 0159 anti-human HLA-DR	Biolegend totalseq B	307661
TotalSeq™-B 0161 anti-human CD11b	Biolegend totalseq B	301357
TotalSeq™-B 0171 anti-human/mouse/rat CD278 (ICOS)	Biolegend totalseq B	313551
TotalSeq™-B 0355 anti-human CD137 (4-1BB)	Biolegend totalseq B	309837
TotalSeq™-B 0386 anti-human CD28	Biolegend totalseq B	302961
TotalSeq™-B 0390 anti-human CD127 (IL-7Rα)	Biolegend totalseq B	351354
TotalSeq™-B 0391 anti-human CD45	Biolegend totalseq B	304066
TotalSeq™-B 0392 anti-human CD15 (SSEA-1)	Biolegend totalseq B	323051

Supplementary Table 3: List of TotalSeq antibodies used to capture surface marker expression with CITE-seq assay.

**Major Class**	**T-cell or Natural Killer cell**								**Plasma Cell**
Cell Type	CD4+ T-Cell	CD4+ Naïve T-Cell	CD4 + Memory T-Cell	CD4+ Regulatory T-Cell	CD8+ T-Cell	CD8+ Exhausted T-Cell	CD8+ Effector T-Cell	Nk	Plasma Cell
Citations	van Galen et al., 2019	van Galen et al., 2019, Wang et al., 2022	van Galen et al., 2019, Wang et al., 2022	van Galen et al., 2019, Wang et al., 2022	van Galen et al., 2019	van Galen et al., 2019, Galletti G. et al., 2020, Hornburg M., et al., 2021	van Galen et al., 2019, Wang et al., 2022	van Galen et al., 2019	van Galen et al., 2019
Markers	CD4	CD3D/E	CD3D/E	CD3D/E	CD3D/E	CD3D/E	CD3D/E	FCER3A	JCHAIN
	CD3D/E	CD4	CD4	CD4	CD8A	TIGIT	CD8A	SLAMF7	MZB1
		SELL	IL7R	FOXP3	IL7R	EOMES	PRF1	PRF1	SDC1
		TCF7	**CD45RO (ADT)**	TIGIT	Low GZMK, GZMB	VSIR	GNLY	GNLY	IGLC2/3
		CCR7		CTLA4	Low cytolytic	**PD1 (ADT)**	GZMB	GZMB	IGKC
		**CD45RA (ADT)**		**CD45RO (ADT)**	Low chemokine markers		GZMK Low	NKG7	IGHA1
					Low exhaustion markers			KLRB1	
					**CD45RO (ADT)**			CD3D/E Negative	
								CD4/8 Negative	

**Major Class**	**Myeloid Lineage**							**Erythroid**	
Cell Type	M1 Macro CD14+	CD1c DC	CD16+ Monocyte	Monocyte	M2 Macro	MDSC	GMP	Erythrocyte	Erythroblast
Citations	van Galen et al., 2019, He et al., 2019, Khan et al., 2022	van Galen et al., 2019	Ravenhill et al., 2020	van Galen et al., 2019	van Galen et al., 2019, He et al., 2019, Khan et al., 2022	van Galen et al., 2019, Kotze et al., 2022, Zhao et al., 2021	van Galen et al., 2019	van Galen et al., 2019	van Galen et al., 2019
Markers	CD14 High	CD1c	FCER3A	CD14	CD163	CD14	AZU1	HBA/B/D	HBA/B/D
	CD68	CLEC10A		S100A8	C1QA	ARG1	MPO	ARG1	CA1
	S100A8	MHC-II High		S100A9		ITGAM	ELANE	CA1	MKI67
	S100A9						MS4A1	FOXO3	TOP2A
	VCAN						MKI67		S100A6
	ITGAX								S100A4
	CD74								
	CTSS								

Major Class	B-Cell				Progenitor/Other		
Cell Type	Pro B-Cell	Pre B-Cell	Naïve B-Cell	Memory B-Cell	pDC	HSC	Stromal
Citations	Lee et al., 2021	Lee et al., 2021	Lee et al., 2021	Lee et al., 2021	van Galen et al., 2019		
Markers	RAG1	CD79A/B	CD79A/B	CD79A/B	GZMB	CD34	CXCL12
	RAG2	MS4A1 LOW	MS4A1	MS4A1	JCHAIN	No CD14	APOE
	IGLL1	IGLL1 - LOW		IGHM	CLEC10A	No CD3D/E	
	DNTT	Light Chain present		CD27	IRF8	No CD79A/B	
	VPREB1			VCAM1		No IGLL1	
						No DNTT	
						No HBA/B/D	
						MKI67	
						TOP2A	
						CENPF/B	

Supplementary Table 4: Table of various cell type markers used to annotate different cell type.
